# Magnetization and Polarization of Coupled Nuclear Spins Ensembles at High Magnetic Fields

**DOI:** 10.1002/cphc.202500092

**Published:** 2025-05-19

**Authors:** Danila A. Barskiy, Andrey N. Pravdivtsev

**Affiliations:** ^1^ Institut für Physik Johannes Gutenberg Universität Mainz 55128 Mainz Germany; ^2^ Helmholtz Institut Mainz 55128 Mainz Germany; ^3^ Section Biomedical Imaging (SBMI)Molecular Imaging North Competence Center (MOIN CC)Department of Radiology and Neuroradiology University Hospital Schleswig‐Holstein (UKSH) Kiel University Am Botanischen Garten 14 24118 Kiel Germany

**Keywords:** coupled spin systems, density matrix, magnetic resonance, magnetization, spin ensembles

## Abstract

In nuclear magnetic resonance (NMR), the bulk magnetization of a sample is commonly assumed to be proportional to spin polarization, with each spin of the same type contributing equally to the measured signal. Herein, the high‐field theorem for general spin‐*I* systems (where *I* is the spin quantum number): the total measurable NMR signal remains unaffected by the grouping of spins into equivalent units (e.g., molecules) is proved, provided the system is at thermodynamic equilibrium in the high field limit ($\hbar \omega_{0}\gg |H_{\text{spin}-\text{spin}}|$, where $\omega_{0}$ is the Larmor frequency and $\left|H_{\text{spin}-\text{spin}}\right|$ characterizes internal spin–spin interactions). The results are derived using both magnetization equations and density matrix formalism. The theorem, however, does not extend to conditions far from thermodynamic equilibrium (e.g., hyperpolarization), NMR of solids in the regime when quadrupolar or dipole–dipole interactions are not negligible, and zero‐ to ultralow‐field NMR. Three educational problems designed to deepen understanding of the material in classroom settings are also presented. This work reinforces established principles in magnetic resonance but also highlights areas for further exploration.

## Graphical abstract

Herein, the magnetization of spins at high magnetic fields using various theoretical approaches is analyzed. The theorem is rigorously proved that the magnetization of individual spins is equivalent to the magnetization of coupled spins within a molecule.

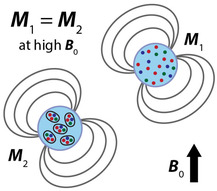

## 1 Introduction

Spin polarization is an important property of spin ensembles. Polarization corresponds directly to the measured signal in experimental techniques like nuclear magnetic resonance (NMR),^[^ ^1^ ^]^ its imaging version, magnetic resonance imaging, and electron paramagnetic resonance.^[^ ^2^, ^3^ ^]^ At a typical magnetic field of 1 T and room temperature, the polarization of ^1^H nuclei is on the order of 10^−5^ (0.001%). Because of the direct proportionality of magnetic resonance signal to polarization, approaches to increase polarization (to create the so‐called “hyperpolarization”) are actively being developed.^[^ ^4^ ^]^ In various textbooks, scientific reviews, and peer‐reviewed publications,^[^ ^5^ ^]^ polarization is typically defined for an ensemble of uncoupled spin‐½ particles. By particle, we mean either individual nuclei or a collection of them. For example, an ensemble of nuclear spins in a molecule with total spin‐*J* can be referred to as spin‐*J* quasiparticle. For such a two‐level system, polarization along the direction of the magnetic field ($P_{z}$, superscript {1/2} indicates spin quantum number) is readily defined by the following equation:

(1) $$P_{z}^{\left\{1/2\right\}}=\frac{n_{\alpha }-n_{\beta }}{n_{\alpha }+n_{\beta }}$$

Here, $n_{\alpha }$ and $n_{\beta }$ are populations of the spin states that correspond to spin orientations along the magnetic field (*α*) and in the opposite direction (*β*), respectively. This article will focus on analyzing nuclear spin ensembles and corresponding polarization values.

**Figure 1:**
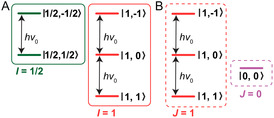
Energy‐level diagrams for A) spin‐½ and spin‐1 particles and B) two equivalent spins‐½ (allowed NMR transitions are shown with frequency $\nu_{0}=\left|\gamma B_{0}/2\pi \right|$ (*γ* > 0 is assumed). Notice that in B), two spins are treated as two semi‐independent spin‐1 and spin‐0 quasiparticles.

It is important to note that despite nuclear spins can be both fermions (half‐integer spins, for example, ^1^H, ^13^C, ^15^N, ^19^F, ^31^P) and bosons (integer spins, for example, ^2^D, ^6^Li, ^14^N, ^36^Cl), in virtually all situations encountered in solution‐state NMR at standard conditions (≈25 °C, ≈1 bar) nuclear wavefunctions do not overlap, and Boltzmann statistics can be applied for both types of particles.^[^ ^6^, ^7^ ^]^ Therefore, by defining the Boltzmann coefficient as a ratio of nuclear energy‐level splitting due to interaction of spins with the external magnetic field $B_{z}$ (**Figure** **1**) and an available thermal energy in the system, $B=\gamma \hbar B_{z}/k_{B}T$ (where *ℏ* and $k_{B}$ are Plank's and Boltzmann's constants, respectively), polarization of the ensemble of noninteracting spins‐½ ($P_{z}^{\left\{1/2\right\}}$) at thermodynamic equilibrium follows from Equation (1):

(2) $$P_{z}^{\left\{1/2\right\}}=\frac{e^{B/2}-e^{-B/2}}{e^{B/2}+e^{-B/2}}=\tanh(B/2)$$

At thermodynamic equilibrium under high temperature (HT) approximation (i.e., when B≪1), $e^{\pm B/2}\approx 1\pm B/2$; thus,

(3) $$P_{z}^{\left\{1/2\right\}}≅\frac{1}{2}B$$

One can readily see that for a specific case of isolated spins‐½, overpopulation of one level with respect to another creates polarization, i.e., a specific orientation of a vector quantity in space. Therefore, restricting “polarization” and “hyperpolarization” to describe vector quantities seems reasonable. However, Bengs and Levitt presented compelling arguments as to why this terminology can also be used to represent other spin orders.^[^ ^8^ ^]^ At the same time, a specific distribution of populations could also have different names, such as alignment, scalar order, population imbalance, latent polarization, quadrupolar order, and so on.^[^ ^8^, ^9^, ^10^, ^11^ ^]^

Our article is motivated by trying to answer two seemingly simple questions: 1) What is polarization for a general case of spin‐*I* particles at thermal equilibrium? 2) Does the magnetic equivalence of spins in molecules nonlinearly affect the total magnetization of the system at thermal equilibrium?

In the following, we will consider these questions and demonstrate several approaches to finding the answers. We constrain ourselves to situations of nuclear spin systems at thermodynamic equilibrium and high magnetic fields, i.e., when Zeeman interactions are much larger than any other interactions in the system, $\gamma \hbar B_{z}\gg \|H_{\text{spin}-\text{spin}}\|$. In this case, one can neglect all interactions except Zeeman in the spin state population distribution.

## 2 Results and Discussion

### 2.1 Formal Definition of Polarization from Bulk Magnetization

Magnetic field $\vec{B}$ in a material is typically defined as a sum of two fields, $\vec{H}$ and $\vec{M}$ such that $\vec{B}=\mu_{0}\left(\vec{H}+\vec{M}\right)$, where $\vec{M}$ is magnetization (the dimension of the components in meter–kilogram–second units is [A/m]). When referring to magnetization in this work, we consider it to be created by nuclear spins. Assuming homogeneous magnetization in volume *V*, the magnetic moment $m_{z}$ of the whole sample (units [A m^2^]) is defined as $m_{z}^{\left\{I\right\},N}=M_{z}\cdot V$. The superscript “{I},N” indicates that magnetization is generated by *N* spin‐*I* particles:

(4) $$m_{z}^{\{I\},N}=N\cdot \langle \mu_{z}^{I}\rangle =N\cdot \gamma \hbar \langle I_{z}\rangle$$

Here, $\left\langle \mu_{z}^{I}\right\rangle$ is an average magnetic moment per spin‐*I* measured along the *z*‐axis. For a single spin, $\mu_{z}^{I}$ is associated with spin projection $I_{z}$ and its behavior is theoretically described by a wavefunction $|\psi \rangle =|I,I_{z}\rangle$. Equation (4) can be considered “Lemma 0” of this work; while it is trivial, it formally defines $\left\langle \mu_{z}^{I}\right\rangle$.

Since for a fully (100%) polarized system of spin‐*I* particles, the maximum magnetic moment is reached when all spins have their maximal projection ${\left\langle \mu_{z}^{I}\right\rangle }_{\text{max}}=\gamma \hbar I$,

(5) $$m_{z,\text{max}}^{\{I\},N}=N\cdot \gamma \hbar I$$

For a fully polarized spin‐*I* system, the state with projection *I* (or −I) has a population of 1. Note that spin‐0 particles have no sizable projection of spin in any direction, hence, they do not induce magnetization. In all other cases, it is reasonable to define polarization, $P_{z}^{\left\{I\right\}}$, as a *z*‐component of a vector quantity proportional to magnetization normalized to its maximal value:^[^ ^12^ ^]^

(6) $$P_{z}^{\left\{I\right\}}=\frac{m_{z}^{\{I\},N}}{m_{z,\text{max}}^{\{I\},N}}=\frac{\left\langle I_{z}\right\rangle }{I}=\frac{1}{I}\sum_{I_{z}=I}^{-I}I_{z}\cdot \rho_{I_{z}}$$

Here, $\rho_{I_{z}}$ is a fraction of spins with projection $I_{z}$; summation runs over (2I+1) states, from |I,I⟩ to |I,−I⟩. Note that if magnetization was measured in directions other than *z*, the basis would have to change accordingly. By using a density matrix operator, $\hat{\rho }$, instead of wave functions, the same information about the system can be obtained:^[^ ^10^ ^]^

(7)

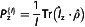




We assume that the density matrix is normalized, i.e., $\text{Tr}\left(\hat{\rho }\right)=1$. In cases where the density matrix is diagonal in the basis corresponding to the measurement basis, Equations (6) and (7) are identical—otherwise, Equation (7) is more general than Equation (6) as it is valid in any basis set. Since we excluded cases of zero spin, one may notice that $P_{z}^{\left\{I\right\}}$ can be always factorized out of the average magnetic moment per particle; therefore, it represents an ensemble property and does not depend on *N*.

To summarize and generalize Equation (4) to the ensemble of different spins, we conclude that the magnetic moment of a sample can always be represented as a product of the number of particles times maximal magnetic moment per particle times polarization of the ensemble:

(8) $$m_{z}^{\{I_{k}\},N_{k}}=N_{k}\cdot \langle \mu_{z}^{I_{k}}\rangle \;=N_{k}\cdot (\gamma_{k}\hbar I_{k})\cdot P_{z}^{\left\{I_{k}\right\}}$$

Here, $I_{k}$ is a quantum number corresponding to the *k‐*th spin type (1/2, 3/2, etc.) with gyromagnetic ratio $\gamma_{k}$ and $N_{k}$ is the number of *k‐*th spins in the sample. A “particle” can represent a spin, a molecule, or even a specific realization of total spin made by a group of nuclei in the molecule (quasiparticles).

### 2.2 Thermodynamic Equilibrium, High‐Field, and High‐Temperature Approximations

In thermodynamic equilibrium, a partition function *ℤ* and temperature *T* are defined via average energy ⟨E⟩ of the system such that

(9) ⟨E⟩=−kTlnZ

Since for spins $\langle E\rangle =-\left(\gamma \hbar I\right)P_{z}^{\left\{I\right\}}B_{z}$, polarization can be deduced from the partition function:

(10) $$P_{z}^{\left\{I\right\}}=\frac{1}{I}\frac{d\ln Z}{dB}$$

If a spin system is in a state that is far from thermodynamic equilibrium, Equations ((9), (10)) cannot be used.

In this work, we will only consider high‐field (HF) approximation: the case when the interaction of a spin‐*I* system with the external magnetic field dominates all other spin–spin interactions, $\gamma \hbar B_{z}\;\gg \|H_{\text{spin}-\text{spin}}\|$. Under this assumption, the partition function has a simple exponential structure:

(11) $$Z\approx \sum_{I_{z}}e^{BI_{z}}$$

In the case of additional HT approximation, a further condition is set: the thermal energy of the system is larger than gaps between spin energy levels: $kT\gg \gamma \hbar B_{z}\;\gg \|H_{\text{spin}-\text{spin}}\|$ and exponentials can be simplified using Taylor expansion:

(12) $$Z^{\text{HT}}=\sum_{I_{z}}(1+BI_{z})+O(B^{2})$$

In the following, we only consider cases of thermodynamic equilibrium and HF approximation and omit additional sub/superscripts to indicate this. When the HT approximation is used, it will be marked with a superscript “HT.”

### 2.3 Polarization of a General Spin‐*I* System

Following Equations ((6), (7)) and/or Equations ((10), (11)), polarization at thermal equilibrium and at high fields can be calculated as

(13) $$P_{z}^{\left\{I\right\}}=\frac{\sum_{I_{z}}I_{z}e^{BI_{z}}}{I\sum_{I_{z}}e^{BI_{z}}}$$

To obtain an analytical expression, one can use known sums of geometric progression (see **Appendix A1**):

(14) $$P_{z}^{\left\{I\right\}}=\frac{1}{I}\left\{\left(I+\frac{1}{2}\right)\text{coth}\left(\left(I+\frac{1}{2}\right)B\right)-\frac{1}{2}\text{coth}\left(\frac{B}{2}\right)\right\}$$

which is known as the Brillouin function, $P_{z}^{\left\{I\right\}}\in \left[-1,1\right]$.^[^ ^12^ ^]^ Under HT approximation (B→0) polarization simplifies to

(15) $$P_{z}^{\{I\},\text{HT}}≅\frac{I+1}{3}B$$

This result can be also obtained directly from Equation (6) considering the population of a state with a projection $I_{z}$ is $\left(1+BI_{z}\right)/\left(2I+1\right)$. Then, using the known equalities for a sum of squares, including the case of half‐integer numbers (**Appendix A2**), Equation (15) is derived.

With this, we answer question (1) laid out in the introduction by finding the polarization of a system of independent spins‐*I* given the HF approximation.

### 2.4 Magnetization of an Arbitrary Spin System

Having found the polarization of spin‐*I* at thermal equilibrium, we will now see if it is different when it is one of many coupled equivalent spins. This we will do in two steps, formulating two lemmas and then a general theorem for the magnetization of spins.

**Lemma 1**: Under high‐field conditions, the magnetization of a system consisting of *k* different types of magnetically inequivalent spins is a sum of their individual magnetizations:

(16) $$m_{z}^{\{I_{1}I_{2}\ldots I_{k}\},N}=\sum_{r=1}^{k}m_{z}^{\{I_{r}\},N_{r}}$$

Here, $N_{k}$ is a number of spins of the type k, and the sum runs over all possible *k* types of spins, $N=\sum_{r=1}^{k}N_{r}$. The syntaxis above implies that $m_{z}^{\left\{I_{r}\right\},N_{r}}$ corresponds to the magnetic moment originating from only one type of spins, spin‐$I_{r}$ particles. Proof comes from the fact that the magnetic field is an additive property of its sources; this is generally true when the field originating from each spin is small compared to the external magnetic field. Otherwise, one should consider additional field amplification.

**Lemma 2**. Under high‐field thermal equilibrium conditions, the magnetization of 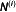 molecules of the type (i)—each containing $n^{\left(i\right)}$ magnetically equivalent spins‐*I* equals to 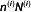 magnetizations of noninteracting spins‐*I*:
(17)

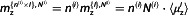




The proof is given in **Appendices A4‐5** using an approach involving classical magnetization and **Appendix A6** using the density matrix treatment. Equation (17) can also be seen as an extension to the indexing notation introduced above (Equation 8). The collorary of the Lemma 2 is that the grouping of spins in molecules does not affect the total magnetization of the system, i.e., $m_{z}^{\{n\times I\},N/n}=m_{z}^{\{I\},N}=N\cdot (\gamma \hbar I)\cdot P_{z}^{\left\{I\right\}}$. This answers the question (2) laid out in the introduction.

**Theorem**. Magnetization of any spin system at thermal equilibrium and at a high field $\left(\gamma \hbar B_{z}\gg \|H_{\text{spin}-\text{spin}}\|\right)$ is an additive quantity that can be expressed as
(18)

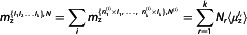




Here, $n_{k}^{\left(i\right)}$ is a number of spins of the type *k* in molecules of the type (i). Summation over all molecules gives a number 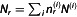 of the *r‐*th type of spins in the system $\langle \mu_{z}^{I_{r}}\rangle =(\gamma \hbar I_{r})\cdot P_{z}^{\left\{I_{r}\right\}}$.

**Figure 2:**
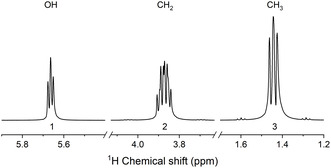
^1^H NMR spectrum of ethanol (CH_3_CH_2_OH) measured at 9.4 T and 293 K. The implication of the **theorem** (Equation (18)) is that the magnetization of each chemical group (being proportional to the integral under the corresponding spectral lines) in high‐field NMR spectra is linearly proportional to the number of protons, irrespective of their magnetic equivalence. The sample is 600 μL of ethanol (>99.5%, 83813.360, TechniSolv) in a 5 mm NMR tube.

The **theorem** follows directly from Lemmas 1 and 2. To summarize, one can see that at thermal equilibrium in high field, it is always possible to divide total magnetization into individual contributions originating from different spin types and/or different molecules, irrespective of their magnetic equivalence (**Figure** **2**).

While these properties (Equations (16)−(18)) may seem obvious, it is essential to realize that conditions of their applicability do not hold at low fields (i.e., when internal spin–spin interactions dominate Zeeman interactions) and far from thermodynamic equilibrium.

This **theorem** has profound implications in conventional high‐field NMR experiments. Indeed, one can feel safe calculating concentrations of different compounds by comparing corresponding NMR signals. At the same time, the ratio of individual signals originating from the same molecules equals to the ratio of the number of nuclei corresponding to these signals. For example, the measured ratio of ^1^H NMR signals in ethanol (CH_3_CH_2_OH) is precisely 3:2:1 (Figure 2).

### 2.5 Problems

Until now, we discussed magnetization of spin systems in general cases and came up with a ubiquitously used by NMR spectroscopists fact formulated as the **theorem**. In this section, we prepared a few problems to test understanding of the mathematics behind the **theorem** and to derive it for some specific cases.

#### 2.5.1 Problem #1. Thermal Magnetization of Two Coupled Spins‐½

Compare the magnetic moments of three thermally polarized samples of the same volume and geometry at the high magnetic field. Sample 1 consists of *N* noninteracting spins‐½ particles. Sample 2 consists of N/2 pairs (molecules) of spins‐½. Sample 3 consists of *N* noninteracting spins‐1 particles. Use Equation (8) and (13) for the derivation of magnetizations $m_{z}^{\left\{1/2\right\},N}$, $m_{z}^{\left\{2\times \left(1/2\right)\right\},N/2}$, and $m_{z}^{\left\{1\right\},N}$ without HT approximation. Express $m_{z}^{\left\{1\right\},N}$ through $m_{z}^{\left\{1/2\right\},N}$ for the cases B≫1 and B≪1.

The detailed solution is given in **Appendix 5**.

#### 2.5.2 Problem #2. NMR Signal Intensities for Coupled Spin Systems Using Fermi's Golden Rule

Find NMR signal intensities for the three samples introduced above using Fermi's Golden rule by considering weak continuous wave excitation of NMR transitions. For the Sample 2, compare the total signals for AX and A_2_ systems. Use the Zeeman basis set for the cases of the single spin‐½ and AX pair and S–T basis for the A_2_ system. This problem is instructive for better understanding the connection between spectral lines and NMR energy levels.

The detailed solution is given in **Appendix 6**.

#### 2.5.3 Problem #3. Magnetization of a Coupled Spin System as if it is Comprised of Quasiparticles

Two equivalent spins‐½ can be naturally described using S–T basis. The singlet state corresponds to a quasiparticle with spin‐0 and the triplet states correspond to the quasiparticle with spin‐1. Only spin‐1 particles contribute to the magnetization. Therefore, instead of calculating magnetization for a coupled two spin‐½ system, one could estimate the magnetization of a system made of spin‐1 particles. Here, we propose to: 1) Given HT approximation, calculate the magnetization of *N* pairs of spins‐½ considering that it is comprised of two independent quasiparticles with spin‐0 and spin‐1. 2) Show that this approach of combining magnetization of quasiparticles is possible also without HT approximation.

*Solution hint:* Note that given HT approximation, number of spin‐0 particles is *N*/4 and the number of spin‐1 particles is 3*N*/4.

The detailed solution is given in **Appendix 7**.

### 2.6 Generalized Spin Polarization: Density Matrix Treatment

In the following, we will introduce an alternative formulation of the **theorem**, now derived using the density matrix approach. This method is typically not explained in a sufficient level of detail in physical chemistry classes. Therefore, first, we present a short overview of the main properties of density matrices. Second, we show which properties of the spin system are missing when only magnetization is considered; they appear naturally in the case of the density matrix description.^[^ ^13^ ^]^

The important quality of the density matrix is that $\text{Tr}\left(\hat{\rho }\right)=1$ which means that the probability of the system to be in all available states sums up to 100%. Assuming Boltzmann distribution of populations for nuclear spins at any temperature *T*, the thermal equilibrium density matrix of a system of an arbitrary number of spins described by the Hamiltonian $\hat{H}$ is

(19) $${\hat{\rho }}^{\text{th}}=\frac{e^{-\frac{\hat{H}}{k_{B}T}}}{\text{Tr}\left(e^{-\frac{\hat{H}}{k_{B}T}}\right)}$$

This expression is very similar to the familiar Boltzmann distribution (Equation (13)), but now written in the matrix form. The obvious useful equality of ${\hat{\rho }}^{\text{th}}$ is commutation with the Hamiltonian since $\hat{H}e^{-\frac{\hat{H}}{k_{B}T}}=e^{-\frac{\hat{H}}{k_{B}T}}\hat{H}$. Because we only consider cases of thermodynamic equilibrium, we will omit the superscript “th” when mentioning $\hat{\rho }$ in the following.

A typical form of a nuclear spin Hamiltonian in liquid state at high fields is
(20)

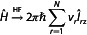

where $2\pi \nu_{r}=\omega_{r}=-\gamma_{r}B$ is Larmor precession frequency and $\gamma_{r}$ is a gyromagnetic ratio for the *r*‐th type of spin. An operator of the *z*‐polarization of *r*‐th spin is
(21)

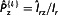




Then, polarization of the *r‐*th type of spins (an expectation value of ${\hat{P}}_{z}^{\left\{I_{r}\right\}}$) is calculated as
(22)

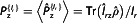




Some authors find it convenient to use normalized irreducible tensors.^[^ ^8^ ^]^ For the spin operator ${\hat{I}}_{rz}$ corresponding irreducible tensor, using Equation A 2.5, is
(23)

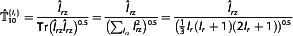




Therefore, polarization can be calculated as
(24)

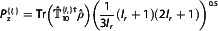




### 2.7 Single Spin‐½

Before going to a deeper analysis of multispin systems, let us first consider a case of independent spin‐½ particles. The corresponding matrix representation of the spin polarization operator in Zeeman basis {|α⟩,|β⟩} is as follows:

(25) $${\hat{I}}_{z}=\frac{1}{2}\left(\begin{array}{cc} 1 & 0\\ 0 & -1 \end{array}\right)$$

For spins other than ½, the matrix representation of the operator ${\hat{I}}_{z}$ is different but, in the Zeeman basis, it is a diagonal matrix with rank (2*I*+1) which consists of possible spin projections from *I* to –*I* with the step of 1. Therefore, one can find that, $\text{Tr}\left({\hat{\text{I}}}_{z}\right)=0\text{,}\;\;\text{Tr}\left({\hat{I}}_{z}{\hat{I}}_{z}\right)=\sum_{I_{z}}I_{z}^{2}=\frac{I(I+1)(2I+1)}{3}$; the latter equality was proved in **Appendix 2**.

Now, using HF Hamiltonian (20), the density matrix given HT approximation is
(26)

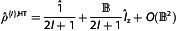

since $\text{Tr}\left(e^{-\frac{\hat{H}}{k_{B}T}}\right)≅\text{Tr}\left(1\right)=2I+1$. Now, one can calculate the polarization of a single spin‐*I:*

(27)

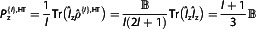




This expression coincides with the result achieved using magnetization (Equation (15)). In the same way, the polarization can be found without HT approximation.

### 2.8 Density Matrix of a Multispin System

To calculate the polarization from an arbitrary size density matrix, we need to define spin operators for a spin system composed of *n* arbitrary spins $\left\{I_{1}I_{2}\ldots I_{n}\right\}$:
(28)

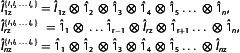

where ⊗ is a direct product, ${\hat{1}}_{r}$ is an identity matrix of the size $\left(2I_{r}+1\right)$ and ${\hat{I}}_{rz}$ is the spin operator of an isolated *r*‐th spin. The identity matrix of this system correspondingly equals to
(29)

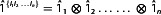




Then, given HT approximation, the thermal equilibrium density matrix of *N* arbitrary spins is
(30)

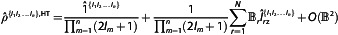




Here, $B_{r}=\gamma_{r}\hbar B/k_{B}T$. From this, polarization of any spin‐$I_{r}$ in an arbitrary coupled spin system is
(31)

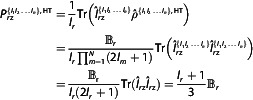




To calculate Equation (31), we used the property of a trace operator with regard to a direct product that allows one to group corresponding matrices: $\text{Tr}\left({\hat{I}}_{rz}^{\left\{I_{1}I_{2}\ldots I_{n}\right\}}{\hat{I}}_{rz}^{\left\{I_{1}I_{2}\ldots I_{n}\right\}}\right)=\text{Tr}\left({\hat{1}}_{1}{\hat{1}}_{1}\right)=\text{Tr}\left({\hat{1}}_{1}{\hat{1}}_{1}\right)\text{Tr}\left({\hat{1}}_{2}{\hat{1}}_{2}\right)\ldots \text{Tr}\left({\hat{I}}_{rz}{\hat{I}}_{rz}\right)\ldots \text{Tr}\left({\hat{1}}_{n}{\hat{1}}_{n}\right)$ and each $\text{Tr}\left({\hat{1}}_{m}{\hat{1}}_{m}\right)=\text{Tr}\left({\hat{1}}_{m}\right)=2I_{m}+1$. Hence, at high magnetic field, polarization of a multispin system with respect to the spin‐*I*_r_ particles is the same as polarization of a system of isolated noninteracting spin‐*I*_r_ particles. This leads to a second and equivalent formulation of the **theorem**, which is now proven with the density matrices.

**Theorem** (alternative formulation): Polarization of a spin‐$I_{r}$ in any spin system at thermal equilibrium and at a high field $\left(\gamma \hbar B_{z}\gg \|H_{\mathrm{spin}-\mathrm{spin}}\|\right)$ equals the polarization of an isolated spin‐$I_{r}$ at the same magnetic field

(32) $$P_{rz}^{\left\{I_{1}I_{2}\ldots I_{n}\right\}}=P_{z}^{\left\{I_{r}\right\}}$$

Considering that $\langle \mu_{z}^{I_{r}}\rangle =\left(\gamma \hbar I_{r}\right).P_{z}^{\left\{I_{r}\right\}}$, and the additive nature of magnetization, the equivalence of two formulations is apparent. Equation (31) proves Equation (32) only at HT approximation, while the general case is considered in **Appendix 8**.

### 2.9 Longitudinal Two‐Spin Order in a Two‐Spin‐½ System

So far, we have considered polarization of a spin. However, in multispin systems, higher orders are also populated. This section will consider a two‐spin‐½ system and find its equilibrium state with higher precision than in Equation (30). Molecular H_2_ or methylene groups of various carbohydrates or amino acids represent typical two‐spin‐½ systems. The density matrix of two spins‐½ up to the second order $\left(O(B^{3})\right)$ is
(33)

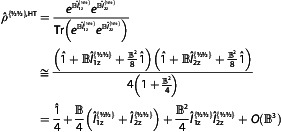




The maximum value of $\left\langle {\hat{I}}_{1z}^{\left\{\text{½½}\right\}}{\hat{I}}_{2z}^{\left\{\text{½½}\right\}}\right\rangle$ equals to ¼, while in general, it is equal to the product of maximum projections of two spins, $I_{1}I_{2}$. Hence, it is reasonable to define polarization of such two‐spin order as

(34) $$P_{rt\text{zz}}^{\left\{I_{1}I_{2}\ldots I_{n}\right\}}=\frac{\left\langle {\hat{I}}_{rz}^{\left\{I_{1}I_{2}\ldots I_{n}\right\}}{\hat{I}}_{tz}^{\left\{I_{1}I_{2}\ldots I_{n}\right\}}\right\rangle }{I_{r}I_{t}}$$

for any spins $I_{r}$ and $I_{t}$ in a multispin system. Using Equations (33) and (34), we can now calculate polarization of such two‐spin order under HT approximation:
(35)

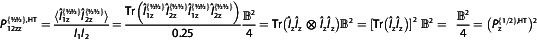




Above, we omitted spin indices after the third equality because only spin‐½ particles are discussed and they have the same spin operators (Equation (25)) and $\text{Tr}\left({\hat{I}}_{z}{\hat{I}}_{z}\right)=\frac{1}{2}$.

Without HT approximation, it can be calculated that
(36)

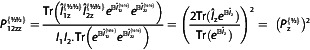




**Figure 3:**
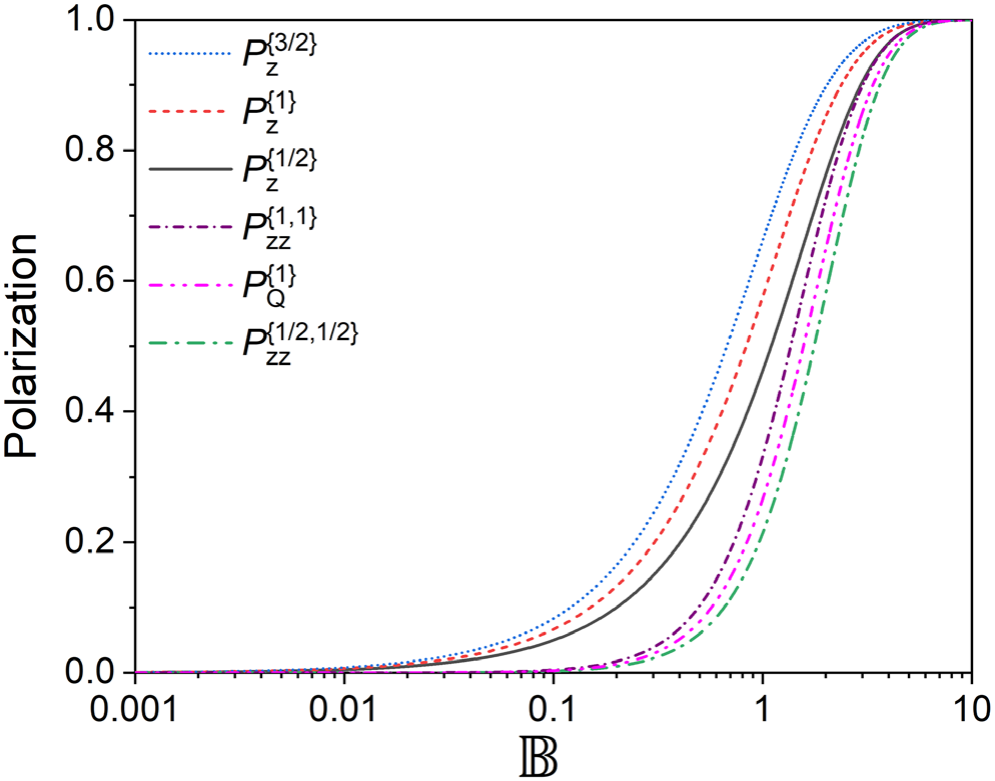
Polarization $P_{z}^{\left\{I\right\}}$ for spins‐½, 1, and 3/2, alignment $P_{Q}^{\left\{I\right\}\;}$ for spin‐1 and $P_{\text{zz}}^{\left\{I,I\right\}}$ for pairs of spin‐½ and spin‐1 systems as a function of B (Boltzmann coefficient).

The first and the third equalities are definitions of polarization, while the second equality is trivially derived using the same idea as in Equation (35): different spins commute and the trace of direct products is the product of traces. Hence, at thermal equilibrium, $P_{12\text{zz}}^{\left\{\text{½½}\right\}}={\left(P_{z}^{\left\{½\right\}}\right)}^{2}$ and combining with Equation (14) it gives a functional dependence of $P_{12\text{zz}}^{\left\{\text{½½}\right\}}$ on B. Both values, $P_{12\text{zz}}^{\left\{\text{½½}\right\}}$ and $P_{z}^{\left\{½\right\}}$, asymptotically reach unity but the two‐spin order is reaching it “slower” (**Figure** **3**).

### 2.10 Density Operator Representation for Multispin Systems

Any operator can be decomposed in terms of its basis states. Irreducible tensors are very common as such a basis in NMR because they are naturally orthonormal. The other approach is based on product operators since they are easy to visualize, and their evolution can be described geometrically. Following Equation (33) and definitions of $P_{z}^{\left\{½\right\}}$ and $P_{12\text{zz}}^{\left\{\text{½½}\right\}}$, the density matrix of two spin‐½ systems can be derived:
(37)

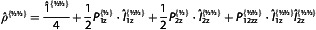




This equation gives the density matrix of the system with a polarization of $P_{1z}^{\left\{½\right\}}$ and $P_{2z}^{\left\{½\right\}}$ and two‐spin order polarization $P_{12\text{zz}}^{\left\{\text{½½}\right\}}$. When thermal equilibrium polarization values are used, the resulting matrix is also at thermal equilibrium.

Analogously, one can obtain that for *N* spins‐½ the density matrix can be then written in the following way:
(38)

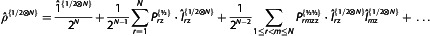




And finally for *N* arbitrary spins, the density matrix is given by the following expression:
(39)

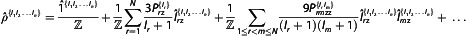

where 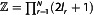. The polarization of other spin orders can be derived and added in the same way if necessary. In the following section, we will derive one more term in the density matrix, which is present for spin‐*I* particles with *I* > ½ and which so far has not been considered by us.

### 2.11 Quadrupolar Polarization of a System with Spin‐*I* (*I* > ½)

In the previous section, we discussed how different spin orders can be populated in multispin systems. Here, we will show that even in a single spin‐*I* system, a state could not be fully described with polarization alone. Specifically, Equation (26) was obtained up to the first order of precision for the magnetic field, $O(B^{2})$. Let us have a look at the same density matrix of a single spin‐*I* with a higher precision using HT approximation:
(40)

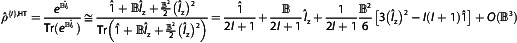




Here, the denominator was simplified as $\text{Tr}\left(\hat{1}+B{\hat{I}}_{z}+\frac{B^{2}}{2}{\left({\hat{I}}_{z}\right)}^{2}\right)=\text{Tr}\left(\hat{1}+\frac{B^{2}}{2}{\left({\hat{I}}_{z}\right)}^{2}\right)=\left(2I+1\right)+\frac{B^{2}}{2}\sum_{I_{z}=I}^{‐I}{\left(I_{z}\right)}^{2}=\left(2I+1\right)\left(1+\frac{B^{2}}{2}\frac{I\left(I+1\right)}{3}\right)$.

This expansion looks very similar to the multipole expansion used, for example, in electrodynamics with dipolar $\left({\hat{I}}_{z}\right)$ and quadrupolar $\left(3{\left({\hat{I}}_{z}\right)}^{2}-I(I+1)\hat{1}\right)$ magnetic moments; the latter term is also often referred to as the “alignment.”^[^ ^12^ ^]^ Another important fact is that for spin‐½ systems ${\left({\hat{I}}_{z}\right)}^{2}=\hat{1}/4$ and, hence, quadrupolar moment equals to zero. One can think of spin‐½ as of purely dipolar nature. In all other cases, the trace of the enclosed in the square brackets operator in Equation (40) is nonzero.

Following the previous normalization concept, the operator of quadrupolar polarization is as follows:

(41) $${\hat{P}}_{Q}^{\{I\}\;}=\frac{3{\left({\hat{I}}_{z}\right)}^{2}-I(I+1)\hat{1}}{I^{2}}$$

The corresponding quadrupole polarization (alignment) is (see derivation in **Appendix 9**):

(42) $$P_{Q}^{\left\{I\right\}}=\frac{2(I+1)}{I}+\frac{3}{2I}+\frac{3}{2\sinh^{2}(\frac{B}{2})}-\frac{3}{I^{2}}\left(I+\frac{1}{2}\right)\text{coth}\left(\left(I+\frac{1}{2}\right)B\right)\text{coth}\left(\frac{B}{2}\right)$$

Unlike $P_{z}^{\left\{I\right\}}$, $P_{Q}^{\left\{I\right\}}$ does not change from −1 to + 1. For example, for spin‐1, $P_{Q}^{\left\{I\right\}}$ can change from −2 to 1. However, at the thermal equilibrium $P_{Q}^{\left\{I\right\}}\in \left[0,1\right]$ disregarding the sign of the gyromagnetic ratio **(**Figure 3).

### 2.12 Is Pure 100% Polarization Possible?

Above, we found the density matrices for two‐spin‐½ and multispin systems which comprise single‐spin polarizations, quadrupolar alignment, and multispin order. For example, for two‐spins‐½, the system in equilibrium is characterized by three values: $P_{1z}^{\left\{½\right\}}$, $P_{2z}^{\left\{½\right\}}$, and $P_{12\text{zz}}^{\left\{\text{½½}\right\}}$ each is in the range from −1 to + 1. If all the values were possible, then the volume of the configuration space would have been 8 (a cube with a side of 2). Below, we will demonstrate that there are constrains on possible polarization values that follow from the fundamental properties of density matrices.

By definition, 1) the density matrix is normalized, $\text{Tr}\left(\hat{\rho }\right)=1$, meaning that the probability of finding the system in any state is 1, 2) it is also a Hermitian operator, $\hat{\rho }={\hat{\rho }}^{†}$, and 3) diagonal elements are non‐negative and do not exceed 1, $1\geq \rho_{r}=\rho_{rr}\geq 0$ (meaning that the probability of finding the system in any state is in the range from 0 to 1).

**Figure 4:**
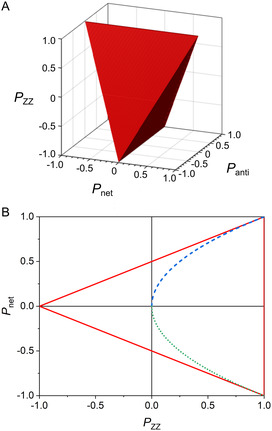
A) The volume of allowed values of polarization for density matrices in units of 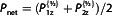,  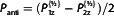 and 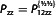 and B) the area of allowed 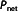 and 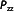 polarization values for 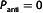. The dashed (blue) and dotted (green) lines indicate polarization values at thermal equilibrium for positive and negative values of the gyromagnetic ratio, repectively. The volume of allowed states in (A) is 4/3, while the nominal available volume is 8. The area of allowed states in (B) is 2, while the nominal available area is 4.

The first two requirements are immediately fulfilled for all the density matrices given above because all operators but $\hat{1}$ are traceless and Hermitian. However, the last requirement puts unexpected restrictions. Considering $1\geq \rho_{r}=\rho_{rr}\geq 0$, the allowed volume for the spin system shrinks from 8 to 4/3 (**Figure** **4**, derived in **Appendix 10**). One should note that in addition to restrictions on populations, there are symmetry restrictions on spin order transfer depending on the spin topology that we do not discuss here.^[^ ^14^, ^15^ ^]^

To understand how it happens in a simple example, let us find the density matrix for two spins‐½ at the state with 100% polarization. The two‐spin order has to be inevitably populated. To rationalize this observation, let us find the spin order representation for the 100% net magnetization. One of the ways is to write density matrices with 100% population of one of the states in the S‐T basis via product operators:^[^ ^16^ ^]^

(43) $${\hat{\rho }}_{T_{+}}^{\left\{\text{½½}\right\}}=|T_{+}\rangle \langle T_{+}|=\frac{{\hat{1}}^{\left\{\text{½½}\right\}}}{4}+\frac{1}{2}\left({\hat{I}}_{1z}^{\left\{\text{½½}\right\}}+{\hat{I}}_{2z}^{\left\{\text{½½}\right\}}\right)+{\hat{I}}_{1z}^{\left\{\text{½½}\right\}}{\hat{I}}_{2z}^{\left\{\text{½½}\right\}},{\hat{\rho }}_{T_{0}}^{\left\{\text{½½}\right\}}=|T_{0}\rangle \langle T_{0}|=\frac{{\hat{1}}^{\left\{\text{½½}\right\}}}{4}+\left({\hat{I}}_{1}^{\left\{\text{½½}\right\}}\cdot {\hat{I}}_{2}^{\left\{\text{½½}\right\}}\right)-2{\hat{I}}_{1z}^{\left\{\text{½½}\right\}}{\hat{I}}_{2z}^{\left\{\text{½½}\right\}},{\hat{\rho }}_{T_{-}}^{\left\{\text{½½}\right\}}=|T_{-}\rangle \langle T_{-}|=\frac{{\hat{1}}^{\left\{\text{½½}\right\}}}{4}-\frac{1}{2}\left({\hat{I}}_{1z}^{\left\{\text{½½}\right\}}+{\hat{I}}_{2z}^{\left\{\text{½½}\right\}}\right)+{\hat{I}}_{1z}^{\left\{\text{½½}\right\}}{\hat{I}}_{2z}^{\left\{\text{½½}\right\}},{\hat{\rho }}_{S}^{\left\{\text{½½}\right\}}=|S\rangle \langle S|=\frac{{\hat{1}}^{\left\{\text{½½}\right\}}}{4}-\left({\hat{I}}_{1}^{\left\{\text{½½}\right\}}\;\cdot {\hat{I}}_{2}^{\left\{\text{½½}\right\}}\right)$$

where $\left({\hat{I}}_{1}^{\left\{\text{½½}\right\}}\cdot {\hat{I}}_{2}^{\left\{\text{½½}\right\}}\right)={\hat{I}}_{1x}^{\left\{\text{½½}\right\}}{\hat{I}}_{2x}^{\left\{\text{½½}\right\}}+{\hat{I}}_{1y}^{\left\{\text{½½}\right\}}{\hat{I}}_{2y}^{\left\{\text{½½}\right\}}+{\hat{I}}_{1z}^{\left\{\text{½½}\right\}}{\hat{I}}_{2z}^{\left\{\text{½½}\right\}}$. All these states have $P_{\text{anti}}=\left(P_{1z}^{½}-P_{2z}^{½}\right)/2=0$; ${\hat{\rho }}_{S}$ and ${\hat{\rho }}_{T_{0}}$ have $P_{\text{net}}=\left(P_{1z}^{½}+P_{2z}^{½}\right)/2=0$ and $P_{\text{zz}}=-1$; ${\hat{\rho }}_{T_{+}}$ has $P_{\text{net}}=1$ and $P_{\text{zz}}=1$; ${\hat{\rho }}_{T_{-}}$ has $P_{\text{net}}=-1$ and $P_{\text{zz}}=1$. The last two states are in the corners of the allowed space for $P_{\text{zz}}=1$ (Figure 4B).

The density matrix of the system with only 100% polarization ($P_{\text{net}}=1$, $P_{\text{anti}}=0$, $P_{\text{zz}}=0$) would be as follows:

(44) $${\hat{\rho }}_{100\%}^{\left\{\text{½½}\right\}}=\frac{\hat{1}}{4}+\frac{1}{2}\left({\hat{I}}_{1z}^{\left\{\text{½½}\right\}}+{\hat{I}}_{2z}^{\left\{\text{½½}\right\}}\right)=\frac{3|T_{+}\rangle \langle T_{+}|+|T_{0}\rangle \langle T_{0}|+|S\rangle \langle S|-|T_{-}\rangle \langle T_{-}|}{4}$$

It is clear that such a state would require a negative population of $|T_{-}\rangle$ which is not feasible. However, the ± 100% polarization is possible and given by ${\hat{\rho }}_{T_{\pm }}$ (Equation (43)) where both $\left|P_{\text{net}}\right|$ and $P_{\text{zz}}$ are maximized.

From Figure 4B, it follows that the highest achievable $P_{\text{net}}$ given $P_{\text{anti}}=P_{\text{zz}}=0$ is only 50%: ${\hat{\rho }}_{50\%}^{\left\{\text{½½}\right\}}=\frac{\hat{1}}{4}+\frac{1}{4}\left({\hat{I}}_{1z}^{\left\{\text{½½}\right\}}+{\hat{I}}_{2z}^{\left\{\text{½½}\right\}}\right)=\frac{2|T_{+}\rangle \langle T_{+}|+|T_{0}\rangle \langle T_{0}|+|S\rangle \langle S|}{4}$. No polarization $\left(P_{\text{net}}=0\right)$ is allowed for $P_{\text{zz}}=-1$.

### 2.13 Special Case: Polarization of H_2_ Molecules

Here, we would like to discuss one peculiar case where molecular degrees of freedom can have an effect on nuclear spin populations using an example of molecular hydrogen. Hydrogen (H_2_) exists under normal conditions in two spin isomeric forms called parahydrogen (*p*H_2_) and orthohydrogen (*o*H_2_). Parahydrogen has a total nuclear spin‐0 while *o*H_2_ has a total spin‐1 resulting in the degeneracy of 1 and 3, respectively. At room temperature conditions, the *ortho*–*para* ratio is close to 3. The ratio at an arbitrary temperature can be calculated using Boltzmann distribution for the rotational levels characterized by a rotational quantum number *J* (magnetic field *B* = 0 as indicated by superscript):

(45) $$\frac{n_{pH_{2}}^{0,T}}{n_{oH_{2}}^{0,T}}=\frac{\sum_{J=2n}(2J+1)\exp(-J(J+1)\theta_{R}/T)}{3\sum_{J=2n+1}(2J+1)\exp(-J(J+1)\theta_{R}/T)}$$

Here, $E_{J=1}-E_{J=0}=2\theta_{R}$ with $\theta_{R}≅87.6$ K.^[^ ^17^ ^]^

The singlet–triplet transitions are forbidden for H_2_ under normal conditions. Paramagnetic materials such as charcoal, iron (III) oxide,^[^ ^18^, ^19^ ^]^ or metal–organic frameworks (MOFs)^[^ ^20^ ^]^ can convert *p*H_2_ to *o*H_2_. Let us now find the thermal equilibrium polarization of H_2_ for a general case in the presence of magnetic field. Since $n_{S}^{B,T}=n_{S}^{0,T}=n_{pH_{2}}^{0,T}$, $n_{T_{\pm }}^{B,T}=n_{T_{\pm }}^{0,T}e^{\pm B}$, $n_{T_{0}}^{B,T}=n_{T_{0}}^{0,T}$ and $n_{T_{+,0,-}}^{0,T}=\frac{n_{oH_{2}}^{0,T}}{3}$, then

(46) $$P_{z}^{\left\{H_{2}\right\}}=\frac{n_{T_{+}}^{B,T}-n_{T_{-}}^{B,T}}{n_{T_{+}}^{B,T}+n_{T_{0}}^{B,T}+n_{T_{-}}^{B,T}+n_{S}^{B,T}}=\frac{e^{B}-e^{-B}}{e^{B}+1+e^{-B}+3\frac{n_{pH_{2}}^{0,T}}{n_{oH_{2}}^{0,T}}}$$

For B≪1, Equation (46) can be simplified to

(47) $$P_{z}^{\left\{H_{2}\right\}}≅\frac{2B}{3\left(1+\frac{n_{pH_{2}}^{0,T}}{n_{oH_{2}}^{0,T}}\right)}$$

where the fraction in denominator is given by Equation (45) which at room temperature is close to 1/3, hence:

(48) $$P_{z}^{\{H_{2}\},\text{HT}}≅\frac{1}{2}B=P_{z}^{\{1/2\},\text{HT}}$$

Thus, molecular hydrogen has the same polarization as atomic hydrogen, and an ensemble of hydrogen molecules in equilibrium at high field gives the same NMR signal as an ensemble of protons.

**Figure 5:**
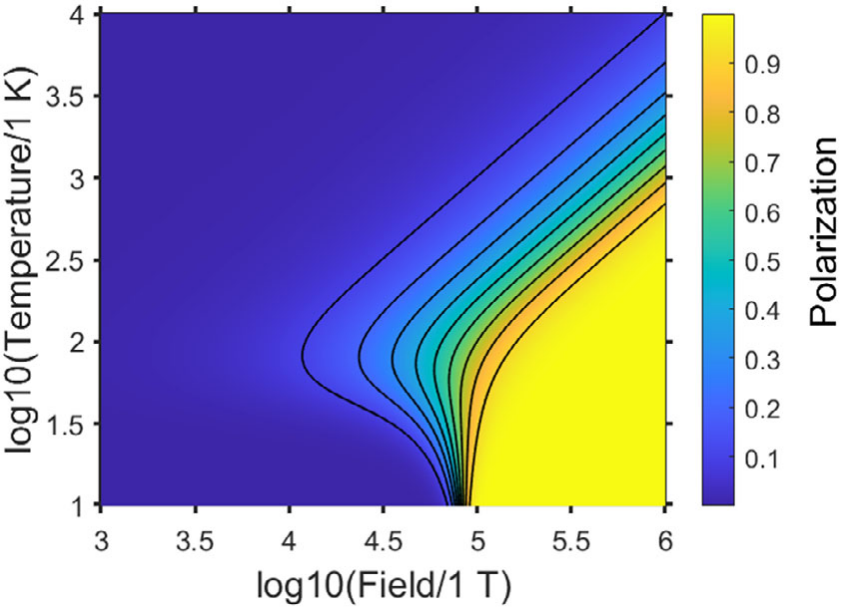
The equilibrium polarization of H_2_ as a function of temperature and magnetic field (Equation 46). At moderate temperatures (10–100 K) and extremely high magnetic fields (≈10^5^ T), 100% polarization is feasible. Solid lines are isolines for polarization values 0.1, 0.2, 0.3, 0.4, 0.5, 0.6, 0.7, 0.8, and 0.9.

It was recently hypothesized that nuclear magnetization of molecular orthohydrogen may be a reason for the magnetism of Jovian planets.^[^ ^21^ ^]^ We analyzed achievable hydrogen polarization levels as a function of magnetic field and temperature using Equation (46) (**Figure** **5**). One can see that it is indeed possible to achieve large (>10%) nuclear polarization at a wide range of temperatures, given that the magnetic field is also large (>10^4^ T). This is reasonable since at such a magnetic field the $|T_{+}\rangle$ state first approaches and then eventually becomes the lowest energy level at $B>2\theta_{R}k_{B}/\gamma \hbar \approx 8.6\cdot 10^{4}$ T, unlike a typically encountered situation where the *para*‐state is the lowest. One can estimate whether or not such a large field could be generated by nuclear spins themselves to further induce polarization of the surrounding spins. Consider answering the following question: What is a magnetic field at the surface of a ball consisting of fully hyperpolarized hydrogen spins? Assuming the density of hydrogen *ρ* and molar mass *M*, the field at the surface of such a ball is

(49) $$B=\frac{\mu_{0}}{2\pi R^{3}}\left(\frac{\gamma \hbar }{2}N\right)=\frac{\mu_{0}\gamma \hbar \rho }{3M}$$

By substituting literature values (mean hydrogen concentration on Jupiter is ρ=1.326 g mL^−1^) into the above expression, one can estimate B≈16 mT irrespective of the size of the ball. Therefore, it is unlikely that the magnetic field generated by nuclear spins alone could induce substantial polarization of surrounding nuclei (self‐induced nuclear ferromagnetism) without other mechanisms of field generation. However, one should note that Equation (45) is based on the assumption of thermal equilibrium which does not work on the scale of Jovian planets. We have not considered possible observable manifestations of multispin orders which could potentially be present as well.

One should also consider that in the core of Jupiter and other gas giants, pressure can be so high that nuclear wavefunctions overlap (it was recently shown to happen above 60 GPa^[^ ^22^ ^]^). Under such conditions, one can no longer assume free molecular rotation as hydrogen becomes liquid (or even solid), and the applicability of Boltzmann statistics breaks down. Therefore, one has to use Fermi–Dirac statistics to estimate polarization which is nontrivial and lies beyond the scope of this article.^[^ ^23^ ^]^

## 3 Conclusion

In this work, we derive equations for magnetization and polarization in multispin systems of arbitrary size and spin quantum numbers, considering cases of thermodynamic equilibrium at high magnetic fields. We formulate two lemmas and the **theorem**, leading to two key conclusions: 1) polarization is independent of the size and topology of the spin system, and 2) magnetization of the system of any spins under high‐field conditions is an additive quantity, irrespective of whether the spins are magnetically equivalent. For educational purposes, we present two alternative formulations of the theorem—one based on classical magnetization and the other using density matrix formalism. To further support understanding, we provide three problems with detailed solutions. Finally, we explore the space of available spin orders in a general two‐spin system and discuss the feasibility of nuclear ferromagnetism in Jovian planets. The results shown here align with previous studies while opening avenues for future research into conditions where the lemmas and **theorem** do not hold, such as in zero‐ to ultralow‐magnetic fields or far from thermodynamic equilibrium.

## Conflict of Interest

The authors declare no conflict of interest.

## Supporting information

Supplementary Material

## Data Availability

Data sharing not applicable ‐ no new data generated
