# Supplementary material for: Magnetization and Polarization of Coupled Nuclear Spins Ensembles at High Magnetic Fields
Source: Chemphyschem. 2025 May 19;26(13):e202500092. doi: 10.1002/cphc.202500092 (PMC12225753; doi:10.1002/cphc.202500092)
Supplement: Supplementary file 1 — Supplementary Material [file CPHC-26-e202500092-s001.pdf]

Supporting information for

Magnetization and Polarization of Coupled Nuclear Spins Ensembles at High Magnetic Fields

Danila A. Barskiy[a,b]* and Andrey N. Pravdivtsev[c]*

[a] Dr. A. Barskiy
Institut für Physik
Johannes Gutenberg Universität Mainz
55128 Mainz, Germany
E-mail: dbarskiy@uni-mainz.de
[b] Helmholtz Institut Mainz
55128 Mainz, Germany
[c] Habil. Dr. A. N. Pravdivtsev
Section Biomedical Imaging, Molecular Imaging North Competence Center (MOIN CC), Department of Radiology and Neuroradiology
University Medical Center Kiel, Kiel University
Am Botanischen Garten 14, 24118, Kiel, Germany
E-mail: andrey.pravdivtsev@rad.uni-kiel.de

Supporting information for this article is given via a link at the end of the document.

Appendices Table of Content

A1. Thermal polarization beyond spin-½ S-2
A2. A sum of squared half-integer numbers is a sum of squared spin projections S-2
A3. Sums involving binomial coefficients S-3
A4. Calculation of magnetization of arbitrary coupled spin-*I* systems S-4
Part 1: Proof of the Lemma 2 for *n* spin-½ S-4
Part 2: Proof of the Lemma 2 for *n* spin-*I* S-5
A5. Solution to the Problem 1 ..... S-7
A6. Solution to the Problem 2 ..... S-8
A7. Solution to the Problem 3 ..... S-10
A8. Calculation of polarization of arbitrary coupled spin-*I* systems. Part 3: Proof of the equivalent formulation of the theorem (eq. 32) S-11
A9. Quadrupolar polarization S-12
A10. Constraints on the elements of the density matrix of two spin-½ S-12

A1. Thermal polarization beyond spin-½

Let us first calculate the sum in the denominator of eq. (13) which is the known sum of a geometric progression:

$$\sum_{I_z=-I}^{I} e^{\mathbb{B}I_z} = \frac{e^{(I+\frac{1}{2})\mathbb{B}} - e^{-(I+\frac{1}{2})\mathbb{B}}}{e^{\mathbb{B}/2} - e^{-\mathbb{B}/2}}. \quad \text{(A1.1)}$$

By differentiating both parts of the above equation over the parameter $\mathbb{B}$, one obtains a numerator:

$$\frac{d}{d\mathbb{B}} \sum_{I_z=I}^{-I} e^{\mathbb{B}I_z} = \sum_{I_z=I}^{-I} I_z e^{\mathbb{B}I_z} = \left(I + \frac{1}{2}\right) \frac{e^{\left(I+\frac{1}{2}\right)\mathbb{B}} + e^{-\left(I+\frac{1}{2}\right)\mathbb{B}}}{e^{\mathbb{B}/2} - e^{-\mathbb{B}/2}} - \frac{1}{2}\left(\frac{e^{\mathbb{B}/2} + e^{-\mathbb{B}/2}}{e^{\mathbb{B}/2} - e^{-\mathbb{B}/2}}\right) \frac{e^{\left(I+\frac{1}{2}\right)\mathbb{B}} - e^{-\left(I+\frac{1}{2}\right)\mathbb{B}}}{e^{\mathbb{B}/2} - e^{-\mathbb{B}/2}}. \quad \text{(A1.2)}$$

Therefore, by finding a ratio of (A1.2) and (A1.1) one obtains

$$\frac{\sum_{I_z=I}^{-I} I_z e^{\mathbb{B}I_z}}{\sum_{I_z=-I}^{I} e^{\mathbb{B}I_z}} = \left(I + \frac{1}{2}\right) \frac{e^{\left(I+\frac{1}{2}\right)\mathbb{B}} + e^{-\left(I+\frac{1}{2}\right)\mathbb{B}}}{e^{\left(I+\frac{1}{2}\right)\mathbb{B}} - e^{-\left(I+\frac{1}{2}\right)\mathbb{B}}} - \frac{1}{2}\left(\frac{e^{\frac{\mathbb{B}}{2}} + e^{-\frac{\mathbb{B}}{2}}}{e^{\frac{\mathbb{B}}{2}} - e^{-\frac{\mathbb{B}}{2}}}\right). \quad \text{(A1.3)}$$

From here, using the hyperbolic cotangent defined as $\coth x = (e^x + e^{-x})/(e^x - e^{-x})$ and dividing the result by I, polarization of a spin-*I* (eq. 14) is obtained. Using Taylor's expansion of hyperbolic cotangent, $\coth x = \frac{1}{x} + \frac{x}{3} - \cdots$, it is straightforward to show that under HT conditions ($\mathbb{B} \ll 1$, $x \ll 1$), indeed, $P_z^{\{I\}} \xrightarrow{\mathbb{B}\to 0} P_z^{\{I\},\mathrm{HT}}$ (eq. 15).

A2. A sum of squared half-integer numbers is a sum of squared spin projections

The sum of the first *N* positive integers is known (sum of arithmetic progression with step 1):

$$\sum_{n=1}^{N} n = \frac{N(N+1)}{2}, \quad \text{(A2.1)}$$

as well as the sum of the squares of the first *N* positive integers:

$$\sum_{n=1}^{N} n^2 = \frac{N(N+1)(2N+1)}{6}. \quad \text{(A2.2)}$$

Therefore, for integer spins, the sum of squares for all possible projections I_z (taking values from $-I$ to $+I$ with the step of 1) is twice this value:

$$\sum_{I_z=\mathrm{I:1:-I}} I_z^2 = \frac{I(I+1)(2I+1)}{3}. \quad \text{(A2.3)}$$

The sum of the half-integer *I*-s can be found as follows. First, we find the sum of squares for *N* odd positive integers:

$$\sum_{n=1}^{N} (2n-1)^2 = 4\sum_{n=1}^{N} n^2 - 4\sum_{n=1}^{N} n + N = 2\frac{N(N+1)(2N+1)}{3} - 2N(N+1) + N = \quad \text{(A2.4)}$$
$$= \frac{N(2N+1)(2N-1)}{3}.$$

If we now divide this expression by 4 and take it twice, we will obtain the requested sum of squares for half-integer numbers. The maximum spin projection *I* corresponds here to $\frac{2N-1}{2}$ and minimum to ½. After substituting $N \to I + 1/2$, one obtains

$$2\sum_{n=1}^{N}\frac{(2n-1)^2}{4} = \frac{N(2N+1)(2N-1)}{6} \to \frac{\left(I+\frac{1}{2}\right)(2I+2)(2I)}{6} = \frac{I(I+1)(2I+1)}{3}. \quad \text{(A2.5)}$$

Hence eq. A2.3 is correct for both integer and half-integer spin quantum numbers.

A3. Sums involving binomial coefficients

Different sums of binominal coefficients appear when one explicitly calculates magnetization of an arbitrary number of spin-½ particles using HT approximation. Here, we will find some sums that will be later used for the derivations of magnetization values. The following three sums involving binomial coefficients $\binom{n}{k} = C_n^k$ are well-known:

$$\sum_0^n C_n^k = 2^n, \quad \text{(A3.1)}$$

$$\sum_{k=0}^{n} kC_n^k = n2^{n-1}, \quad \text{(A3.2)}$$

$$\sum_{k=0}^{n} k^2 C_n^k = (n+n^2)2^{n-2}. \quad \text{(A3.3)}$$

Therefore, the quantity $\sum_{k=0}^{n} C_n^k \left(\frac{n}{2} - k\right)^2$ can be found using (A3.1-3):

$$\sum_{k=0}^{n} C_n^k \left(\frac{n}{2} - k\right)^2 = \sum_0^n C_n^k \left(\frac{1}{4}n^2 - nk + k^2\right) = \frac{1}{4}n^2 \sum_0^n C_n^k - n\sum_{k=0}^{n} kC_n^k + \sum_{k=0}^{n} k^2 C_n^k = \quad \text{(A3.4)}$$
$$= \frac{1}{4}n^2 2^n - \frac{1}{2}n^2 2^n + \frac{1}{4}(n+n^2)2^n = \frac{1}{4}n2^n.$$

Another useful sum involves exponentials encountered when computing magnetization of an arbitrary number of spins-½ without using HT approximation. The following equality follows from the definition of binomial coefficients:

$$\left(e^{\frac{\mathbb{B}}{2}} + e^{-\frac{\mathbb{B}}{2}}\right)^n = \sum_{k=0}^{n} C_n^k e^{\left(\frac{n}{2}-k\right)\mathbb{B}}, \quad \text{(A3.5)}$$

The right part of this equation is a denominator when such magnetization is calculated.

Derivative of the left part of the eq. (A3.5) with respect to $\mathbb{B}$ gives

$$\frac{d}{d\mathbb{B}}\left(e^{\frac{\mathbb{B}}{2}} + e^{-\frac{\mathbb{B}}{2}}\right)^n = \frac{1}{2}n\left(e^{\frac{\mathbb{B}}{2}} + e^{-\frac{\mathbb{B}}{2}}\right)^n \left(\frac{e^{\frac{\mathbb{B}}{2}} - e^{-\frac{\mathbb{B}}{2}}}{e^{\frac{\mathbb{B}}{2}} + e^{-\frac{\mathbb{B}}{2}}}\right). \quad \text{(A3.6)}$$

Derivative of the right part of the eq. (A3.5) with respect to $\mathbb{B}$ gives

$$\frac{d}{d\mathbb{B}}\sum_{k=0}^{n} C_n^k e^{\left(\frac{n}{2}-k\right)\mathbb{B}} = \sum_{k=0}^{n} C_n^k \left(\frac{n}{2} - k\right) e^{\left(\frac{n}{2}-k\right)\mathbb{B}}. \quad \text{(A3.7)}$$

The right part of this equation is a numerator of the corresponding magnetization, e.g. $\frac{n}{2} - k$ corresponds to a spin projection, and C_n^k is the coefficient of this spin projection's degeneracy, as detailed below.

A4. Calculation of magnetization of arbitrary coupled spin-*I* systems

Part 1: Proof of the Lemma 2 for *n* spin-½

We consider only one type of spins in the molecule: *n* is the number of equivalent spins per molecule, and *N* is the total number of molecules. Since the spins are magnetically equivalent, one has to consider the quantum mechanical rules of adding angular momenta. This results in the fact that total spin can take values starting from $J = nI$ down to zero for integer J (**Fig. A1**) or I for a half-integer J. At the high field, total magnetic moment can be computed via projections of the total spin using eq. (13):

$$m_{\mathrm{z}}^{\{n\times I\},N} = N\gamma\hbar \cdot \left(\frac{\sum_{J_{\mathrm{z}}=nI}^{-nI} J_{\mathrm{z}} \cdot G_{n\times I}^{J_{\mathrm{z}}} \cdot e^{\mathbb{B}J_{\mathrm{z}}}}{\sum_{J_{\mathrm{z}}=nI}^{-nI} G_{n\times I}^{J_{\mathrm{z}}} \cdot e^{\mathbb{B}J_{\mathrm{z}}}} \right) \xrightarrow{\mathrm{HT}\ (\mathbb{B}\ll 1)} N\gamma\hbar \cdot \left(\mathbb{B} \frac{\sum_{J_{\mathrm{z}}=nI}^{-nI} J_{\mathrm{z}}^2 \cdot G_{n\times I}^{J_{\mathrm{z}}}}{\sum_{J_{\mathrm{z}}=nI}^{-nI} G_{n\times I}^{J_{\mathrm{z}}}} \right). \quad \text{(A4.1)}$$

Here, $G_{n\times I}^{J_{\mathrm{z}}}$ are coefficients of a generalized Paskal triangle representing multiplicity (degeneracy) of the state with a projection J_{z} of the maximal total spin $J = nI$.

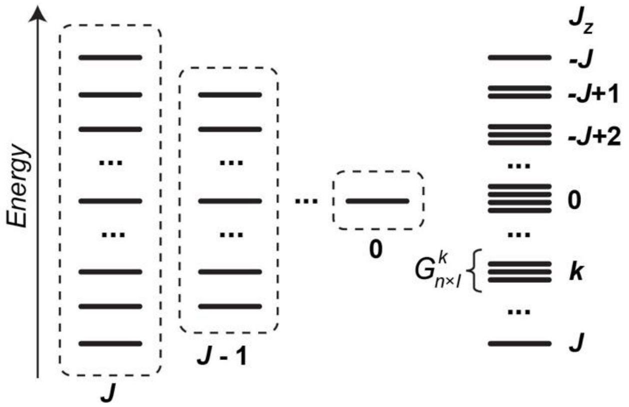

Figure A1. Illustration of the energy-level diagram for an *N*-spin system consisting of *n* spins-*I* per molecule. Left: manifolds corresponding to the total spin *J* = (*nI*), (*J* -1), … , 0 (the total spin-0 exists only when *nI* is an integer). Right: projections of the total spin and corresponding degeneracy of the energy levels, $G_{n\times I}^{J_{\mathrm{z}}}$.

Coefficients $G_{n\times I}^{J_{\mathrm{z}}}$ represent degeneracy of the energy level that corresponds to the total spin projection J_{z}. For spin-½ systems, coefficients $G_{n\times\frac{1}{2}}^{J_{\mathrm{z}}}$ are given by the Pascal triangle [24,25], i.e., they are binomial coefficients $G_{n\times\frac{1}{2}}^{J_{\mathrm{z}}} = C_n^k$, where *n* is the number of spins, $k = n/2 - J_{\mathrm{z}}$ and conversely $J_{\mathrm{z}} = n/2 - k$. The total number of spin projections is therefore $(n+1)$.

Before finding the magnetization in the general case (eq A4.1), let us first consider this simpler case of n spin-½ system with HT approximation. Thirst calculate part in brackets in eq (A4.1):

$$\left(\frac{\sum_{k=0}^{n} C_n^k\left(\frac{n}{2}-k\right)e^{\left(\frac{n}{2}-k\right)\mathbb{B}}}{\sum_{k=0}^{n} C_n^k e^{\left(\frac{n}{2}-k\right)\mathbb{B}}}\right) \approx \mathbb{B}\frac{\sum_{k=0}^{n} C_n^k\left(\frac{n}{2}-k\right)^2}{\sum_{k=0}^{n} C_n^k} = \mathbb{B}\frac{\frac{1}{4}n2^n}{2^n} = \frac{1}{4}n\mathbb{B} = \frac{1}{2}nP_{\mathrm{z}}^{\{1/2\},\mathrm{HT}}. \tag{A4.2}$$

Here, we used the results of eq. (A3.1) and (A3.4) and (3). Therefore eq. (A4.1) can be simplified as,

$$m_{\mathrm{z}}^{\{n\times 1/2\},N} = N\gamma\hbar\cdot\frac{1}{2}nP_{\mathrm{z}}^{\{1/2\},\mathrm{HT}} = n\cdot m_{\mathrm{z}}^{\{1/2\},N}. \tag{A4.3}$$

and the **Lemma 2** is proven for n spin-½ system under HT approximation.

By using eqs. (A3.5-A3.7), without loss of generality for n spin-½ system, part in brackets in eq. (A4.1) can be shown to be equal $\frac{1}{2}nP_{\mathrm{z}}^{1/2}$:

$$\frac{\sum_{k=0}^{n} C_n^k\left(\frac{n}{2}-k\right)e^{\left(\frac{n}{2}-k\right)\mathbb{B}}}{\sum_{k=0}^{n} C_n^k e^{\left(\frac{n}{2}-k\right)\mathbb{B}}} = \frac{\frac{1}{2}n\left(e^{\frac{\mathbb{B}}{2}}+e^{-\frac{\mathbb{B}}{2}}\right)^n\left(\frac{e^{\frac{\mathbb{B}}{2}}-e^{-\frac{\mathbb{B}}{2}}}{e^{\frac{\mathbb{B}}{2}}+e^{-\frac{\mathbb{B}}{2}}}\right)}{\left(e^{\frac{\mathbb{B}}{2}}+e^{-\frac{\mathbb{B}}{2}}\right)^n} = \frac{1}{2}n\cdot\tanh(\mathbb{B}/2) = \frac{1}{2}nP_{\mathrm{z}}^{\{\frac{1}{2}\}}. \tag{A4.4}$$

With this, **Lemma 2** is proven for n spin-½ system at thermal equilibrium at any temperature.

Part 2: Proof of the Lemma 2 for *n* spin-*I*

Now, the same steps could be carried out for n spin-*I* and find the magnetization $m_{\mathrm{z}}^{\{n\times I\},N}$ (eq A4.1). First, we know that the sum of all $G_{n\times I}^{J_{\mathrm{z}}}$ (denominator in eq A4.1) equals to the total number of states in the system:

$$\sum_{J_{\mathrm{z}}=nI}^{-nI} G_{n\times I}^{J_{\mathrm{z}}} = (2I+1)^n. \tag{A4.5}$$

Second, since these coefficients can be obtained by calculating the generalized Pascal triangle for spin-*I* systems [26], they represent relative intensities of lines in high-field NMR spectra of a spin coupled, for example, via *J*-coupling to a group of n spin-*I*.

For example, for n spin-½ these intensities are obtained from the following equation:

$$\left(e^{i\pi Jt}+e^{-i\pi Jt}\right)^n = \sum_{J_{\mathrm{z}}=-n/2}^{n/2} G_{n\times\frac{1}{2}}^{J_{\mathrm{z}}} e^{i\pi Jt J_{\mathrm{z}}}. \tag{A4.6}$$

Exponentials determine frequency offsets of lines in the spectrum, and the numbers before exponents will give us degeneration: for spin-½, coefficients G coinside with binominal coefficients as discussed in previous appendices. Note that in this section J represent spin-spin interaction and J_{z} is a projection of total spin but it is easer to see the difference as spin-spin interaction is always with π on the left to it.

Now, for a general AX_n spin system (where X are spin-*I* particles), the splittings are given by the evolution of the type (analogous to A4.6)

$$\left(\sum_{I_z=-I}^{I} e^{i2\pi J t I_z}\right)^n = \sum_{J_z=-In}^{In} G_{n\times I}^{J_z} e^{i2\pi J t J_z} \quad \text{(A4.7)}$$

One can find $G_{n\times\frac{1}{2}}^{I_z}$ values and generalized Pascal triangles for any spin using multinomial coefficients but we do not need it here. Instead, we need to find only two sums: numerator and denominator in eq. (A4.1). We will start with a simpler case of HT approximation. To make notations shorter we will make a small substitution $i2\pi Jt \to a$. As before, first, we will calculate the denominator of eq. (A4.1) using eq. (A4.7) and putting $a \to 0$:

$$\left(\sum_{I_z=-I}^{I} e^{aI_z}\right)^n = \sum_{J_z=-In}^{In} G_{n\times I}^{J_z} e^{aI_z} \xrightarrow{a\to 0} \left(\sum_{I_z=-I}^{I} 1\right)^n = (2I+1)^n = \sum_{J_z=-In}^{In} G_{n\times I}^{J_z}. \quad \text{(A4.8)}$$

Now, we can find the numerator of eq. (A4.1). To do so, we will differentiate eq. (A4.8) twice and again put limits ($a \to 0$):

$$\frac{d^2}{da^2}\left(\sum_{I_z=-I}^{I} e^{aI_z}\right)^n = n\frac{d}{da}\left[\left(\sum_{I_z=-I}^{I} I_z e^{aI_z}\right)\left(\sum_{I_z=-I}^{I} e^{aI_z}\right)^{n-1}\right] = \quad \text{(A4.9)}$$
$$= n\left(\sum_{I_z=-I}^{I} I_z^2 e^{aI_z}\right)\left(\sum_{I_z=-I}^{I} e^{aI_z}\right)^{n-1} + n(n-1)\left(\sum_{I_z=-I}^{I} I_z e^{aI_z}\right)^2\left(\sum_{I_z=-I}^{I} e^{aI_z}\right)^{n-2} \xrightarrow{a\to 0}$$
$$n\left(\sum_{I_z=-I}^{I} I_z^2\right)\left(\sum_{I_z=-I}^{I} 1\right)^{n-1} = n\frac{I(I+1)(2I+1)}{3}(2I+1)^{n-1} = n\frac{I(I+1)}{3}(2I+1)^n$$

This equals to denominator of eq. (A4.1):

$$\frac{d^2}{da^2}\left(\sum_{I_z=-I}^{I} e^{aI_z}\right)^n = \frac{d^2}{da^2}\left(\sum_{J_z=-In}^{In} J_z^2 G_{n\times I}^{J_z} e^{aJ_z}\right) = \sum_{J_z=-In}^{In} J_z^2 G_{n\times I}^{J_z} e^{aJ_z} \xrightarrow{a\to 0} \sum_{J_Z=-In}^{In} J_Z^2 G_{n\times I}^{J_z} \quad \text{(A4.10)}$$

Hence, now we can combine the equations above and find the part in brackets for magnetization of *n* spins-*I* at HT approximation (eq. A4.1):

Here we used eq. A1.3 from **Appendix A1**. Hence

$$\left(\mathbb{B}\frac{\sum_{J_z=nI}^{-nI} J_z^2 \cdot G_{n\times I}^{J_z}}{\sum_{J_z=nI}^{-nI} G_{n\times I}^{J_z}}\right) = \frac{n\frac{I(I+1)}{3}(2I+1)^n}{(2I+1)^n}\mathbb{B} = n\frac{I(I+1)}{3}\mathbb{B} = nIP_z^{\{I\},\mathrm{HT}}. \quad \text{(A4.11)}$$

This is precisely like eq. A4.2 but for general spin-*I*.

And finally, we can find eq. A4.1 and proof our Lemma 2 in general case without HT approximation. We will use the same ideas as before, but now everywhere, instead of parameter a, we will use $\mathbb{B}$ and easily proof the statement in the general case. The denominator of eq. A4.1 is:

$$\sum_{J_z=-nI}^{nI} G_{n\times I}^{J_z} \cdot e^{J_z\mathbb{B}} = \left(\sum_{I_z=-I}^{I} e^{I_z\mathbb{B}}\right)^n. \quad \text{(A4.12)}$$

And corresponding numerator is its first derivative:

$$\frac{d}{d\mathbb{B}}\sum_{J_z=-nI}^{nI} G_{n\times I}^{J_z} \cdot e^{J_z\mathbb{B}} = \sum_{J_z=-nI}^{nI} G_{n\times I}^{J_z} \cdot J_z e^{J_z\mathbb{B}} = \quad \text{(A4.13)}$$
$$n\sum_{I_z=-I}^{I} I_z e^{I_z\mathbb{B}}\left(\sum_{I_z=-I}^{I} e^{I_z\mathbb{B}}\right)^{n-1} = nI\frac{\sum_{I_z=-I}^{I} I_z e^{I_z\mathbb{B}}}{I\sum_{I_z=-I}^{I} e^{I_z\mathbb{B}}}\left(\sum_{I_z=-I}^{I} e^{I_z\mathbb{B}}\right)^n = nIP_z^{\{I\}}\left(\sum_{I_z=-I}^{I} e^{I_z\mathbb{B}}\right)^n.$$

Here, we used the definition of $P_z^{\{I\}}$ (eq. 13). Now, combining eqs. (A5.8, A5.9 and A4.1) to get the following equation to prove **Lemma 1**.

$$m_z^{\{n\times I\},N} = N\gamma\hbar\cdot\left(\frac{\sum_{J_z=nI}^{-nI} J_z\cdot G_{n\times I}^{J_z}\cdot e^{\mathbb{B}J_z}}{\sum_{J_z=nI}^{-nI} G_{n\times I}^{J_z}\cdot e^{\mathbb{B}J_z}}\right) = N\cdot\gamma\hbar I\cdot n\cdot P_z^{\{I\}} = n\cdot m_z^{\{I\},N}. \quad \text{(A4.14)}$$

That is the quintessence of Lemma 2, and it is now proven in a general case here, demonstrating that magnetization is an additive quantity for equivalent spins of the same molecule.

A5. Solution to the Problem 1

Magnetic moment corresponding to *N* spin-½ particles at HF is given by eqs. (8) and (13):

$$m_z^{\{1/2\},N} = N\cdot\left(\frac{\gamma\hbar}{2}\right)\cdot\left[\frac{e^{\frac{1}{2}\mathbb{B}}-e^{-\frac{1}{2}\mathbb{B}}}{e^{\frac{1}{2}\mathbb{B}}+e^{-\frac{1}{2}\mathbb{B}}}\right]. \quad \text{(A5.1)}$$

If now, instead, we have *N*/2 pairs of interacting spin-½ particles (the total number of spins is still *N*), we first need to change calculation basis accordingly. Two conventional basis sets are used to calculate the pairs of spins. The first is the Zeeman basis: $|1\rangle = |\alpha\alpha\rangle$, $|2\rangle = |\alpha\beta\rangle$, $|3\rangle = |\beta\alpha\rangle$, $|4\rangle = |\beta\beta\rangle$, and the second is the singlet-triplet (S-T) basis (**Figure 1B**): $|0{,}0\rangle = |S\rangle = (|\alpha\beta\rangle - |\beta\alpha\rangle)/\sqrt{2}$, $|1{,}1\rangle = |T_+\rangle = |\alpha\alpha\rangle$, $|1{,}0\rangle = |T_0\rangle = (|\alpha\beta\rangle + |\beta\alpha\rangle)/\sqrt{2}$, $|1,-1\rangle = |T_-\rangle = |\beta\beta\rangle$. Here, $|\alpha\rangle = |1/2\,,1/2\rangle$ and $|\beta\rangle = |1/2\,,-1/2\rangle$, are shorthand notations for spin-½ being parallel and antiparallel to the *z*-axis (here, the first number in the ket is the spin value, and the second number denotes its projection onto the *z*-axis). The S-T basis is a natural choice for two equivalent spins-½ because it separates spin states into two symmetrized manifolds with total spin of 1 and 0, respectively.

Although these two basis sets are different, the application of the eqs. (8-13) will still give the same result. This is because only projections $\mu_z(|\psi\rangle)$ of the total magnetic moment matter, and these projections are the same for Zeeman and S-T basis sets, i.e., {-1, 0, 0, 1}. Hence, one can calculate the magnetization of a pair of spin-½ particles as follows:

$$m_z^{\{2\times(1/2)\},N/2} = \frac{N}{2}\cdot(\gamma\hbar)\cdot\left[\frac{e^{\mathbb{B}}+0+0-e^{-\mathbb{B}}}{e^{\mathbb{B}}+1+1+e^{-\mathbb{B}}}\right] = N\cdot\left(\frac{\gamma\hbar}{2}\right)\cdot\left[\frac{e^{\frac{\mathbb{B}}{2}}-e^{-\frac{\mathbb{B}}{2}}}{e^{\frac{\mathbb{B}}{2}}+e^{-\frac{\mathbb{B}}{2}}}\right] = m_z^{\{1/2\},N}. \quad \text{(A5.2)}$$

Hence, given the same volume and sample geometry, magnetization of samples composed of equivalent spins (such as in H_2 or H_2O molecules) and magnetization of samples composed of magnetically nonequivalent spins of the same concentration at thermal equilibrium and high field coincide. Our Theorem gives an extension to any number of equivalent spins. Note that this equivalence does not require HT approximation but requires thermodynamic equilibrium and HF conditions.

For spin-1 particles, magnetization is as follows

$$m_z^{\{1\},N} = N \cdot (\gamma\hbar) \cdot \left[\frac{e^{\mathbb{B}} + 0 - e^{-\mathbb{B}}}{e^{\mathbb{B}} + 1 + e^{-\mathbb{B}}}\right]. \quad \text{(A5.3)}$$

One can see that the ratio of sample magnetizations as a function of spin quantum number for spin-½ and spin-1 particles is

$$\frac{m_z^{\{1\},N}}{m_z^{\{1/2\},N}} = 2 \cdot \left[1 + \frac{1}{e^{\mathbb{B}} + 1 + e^{-\mathbb{B}}}\right]. \quad \text{(A5.4)}$$

If $\mathbb{B} \gg 1$, $m_z^{\{1\},N} / m_z^{\{1/2\},N} \xrightarrow{\mathbb{B}\to\infty} 2$. Given HT approximation ($\mathbb{B} \ll 1$), however, $m_z^{\{1\},N} / m_z^{\{1/2\},N} \xrightarrow{\mathbb{B}\to 0} m_z^{\{1\},N,\mathrm{HT}} / m_z^{\{1/2\},N,\mathrm{HT}} = 8/3$.

A6. Solution to the Problem 2

Fermi's golden rule determines transition probability per unit time between any two states $|1\rangle$ and $|2\rangle$ [27]:

$$W_{1\to 2} = \frac{2\pi}{\hbar} \left|\langle 2|\hat{V}_{\mathrm{RF}}|1\rangle\right|^2 (n_1 - n_2)\delta(E_2 - E_1 - \hbar\omega). \quad \text{(A6.1)}$$

Here, n_1 and n_2 are populations of the states with energies E_1 and E_2, respectively. The amplitudes of NMR spectral lines are proportional to the corresponding transition probabilities. In the following, we will neglect proportionality coefficients. An on-resonance RF-pulse induces the single-quantum transitions between spin states. The interaction of spins with the RF field is given by the following operator (in the rotation frame of reference):

$$\hat{V}_{\mathrm{RF}} = \gamma\hbar B_1 \hat{I}_{\mathrm{x}}. \quad \text{(A6.2)}$$

For the calculation of intensities, we will use lowering and raising spin operators $\hat{I}_-$ and $\hat{I}_+$ [28]:

$$\hat{I}_{\mathrm{x}} = \frac{\hat{I}_+ + \hat{I}_-}{2}, \hat{I}_{\mathrm{y}} = \frac{\hat{I}_+ - \hat{I}_-}{2i}. \quad \text{(A6.3)}$$

One can find the effect of these operators on spin states with total spin I and projection I_z:

$$\hat{I}_+|I, I_z\rangle = \sqrt{(I - I_z)(I + I_z + 1)}|I, I_z + 1\rangle,$$
$$\hat{I}_-|I, I_z\rangle = \sqrt{(I + I_z)(I - I_z + 1)}|I, I_z - 1\rangle. \quad \text{(A6.4)}$$

Note that $\hat{I}_+|I,I\rangle = 0$ and $\hat{I}_-|I, -I\rangle = 0$. Now we are ready to use the eq. (A6.1) and apply it to the specific cases.

One spin-½ (A). First, one can see that "up" and "down" transitions for one spin-½ (A-system) have the same intensity governed by the following matrix elements:

$$\langle\alpha|\hat{I}_{\mathrm{x}}|\beta\rangle = \langle\beta|\hat{I}_{\mathrm{x}}|\alpha\rangle = \left\langle\beta\left|\frac{\hat{I}_-}{2}\right|\alpha\right\rangle = 1/2. \quad \text{(A6.5)}$$

Therefore, the NMR signal of the A-system consisting of *N* particles is

$$\text{Signal}(N,\text{A}) \sim N \cdot W_{\alpha\to\beta} \sim \left(\frac{\hbar\gamma B_1}{2}\right)^2 (n_\alpha - n_\beta) = N\left(\frac{\hbar\gamma B_1}{2}\right)^2 \left[\frac{e^{\frac{1}{2}\mathbb{B}} - e^{-\frac{1}{2}\mathbb{B}}}{e^{\frac{1}{2}\mathbb{B}} + e^{-\frac{\mathbb{B}}{2}}}\right] \xrightarrow{\mathbb{B}\to 0} N(\hbar\gamma B_1)^2 \cdot \frac{1}{8}\mathbb{B}. \quad \text{(A6.5)}$$

Two spins-½ (A_2). In the system of two equivalent spins (A_2-system), there are four single quantum transitions, but only two of them are allowed:

$$\langle T_-|\hat{I}_x|T_0\rangle = \langle T_0|\hat{I}_x|T_+\rangle = \left\langle T_0\left|\frac{\hat{I}_-}{2}\right|T_+\right\rangle = 1/\sqrt{2}, \quad \text{(A6.6)}$$
$$\langle T_{+1}|\hat{I}_x|S\rangle = \langle T_{-1}|\hat{I}_x|S\rangle = 0.$$

Here, $\hat{I}_x = \hat{I}_{1x} + \hat{I}_{2x}$ is the operator of the *x*-projection of the total spin. The NMR signal from *N*/2 pairs of equivalent spins is a sum of two signals corresponding to the two allowed transitions:

$$\text{Signal}(N/2,\text{A}_2) \sim \frac{N}{2}\left(W_{T_0\to T_-} + W_{T_+\to T_0}\right) \sim \frac{1}{2}\left(\frac{\gamma\hbar B_1}{\sqrt{2}}\right)^2 \left[\left(n_{T_0} - n_{T_-}\right) + \left(n_{T_+} - n_{T_0}\right)\right] = \quad \text{(A6.7)}$$
$$= \frac{N}{4}(\hbar\gamma B_1)^2 \left[\frac{e^{\mathbb{B}} - e^{-\mathbb{B}}}{e^{\mathbb{B}} + 1 + 1 + e^{-\mathbb{B}}}\right] = N\left(\frac{\hbar\gamma B_1}{2}\right)^2 \left[\frac{e^{\mathbb{B}/2} - e^{-\mathbb{B}/2}}{e^{\mathbb{B}/2} + e^{-\mathbb{B}/2}}\right] = \text{Signal}(N,\text{A}).$$

Once again, one can see that the signal from *N*/2 of A_2-systems is the same as from *N* of A-systems.

Two spins-½ (AX). In the case of two chemically nonequivalent spins (AX-system), there are four single-quantum transitions, all of which have the same intensity:

$$\langle\alpha\beta|\hat{I}_x|\alpha\alpha\rangle = \langle\beta\alpha|\hat{I}_x|\alpha\alpha\rangle = \langle\beta\beta|\hat{I}_x|\alpha\beta\rangle = \langle\beta\beta|\hat{I}_x|\beta\alpha\rangle = \frac{1}{2}, \quad \text{(A6.8)}$$

where again, $\hat{I}_x = \hat{I}_{1x} + \hat{I}_{2x}$. Thus, the total NMR signal from *N*/2 pairs of nonequivalent spins (AX-systems) is a sum of four signals from the four allowed transitions:

$$\text{Signal}(N/2,\text{AX}) \sim \frac{N}{2}\left(W_{\alpha\alpha\to\alpha\beta} + W_{\alpha\alpha\to\beta\alpha} + W_{\alpha\beta\to\beta\beta} + W_{\beta\alpha\to\beta\beta}\right) \sim \quad \text{(A6.9)}$$
$$\sim \frac{1}{2}\left(\frac{\gamma\hbar B_1}{2}\right)^2 \left[\left(n_{\alpha\alpha} - n_{\alpha\beta}\right) + \left(n_{\alpha\alpha} - n_{\beta\alpha}\right) + \left(n_{\alpha\beta} - n_{\beta\beta}\right) + \left(n_{\beta\alpha} - n_{\beta\beta}\right)\right] =$$
$$= \frac{N}{4}(\hbar\gamma B_1)^2 \left[\frac{e^{\mathbb{B}} - e^{-\mathbb{B}}}{e^{\mathbb{B}} + 1 + 1 + e^{-\mathbb{B}}}\right] = N\left(\frac{\hbar\gamma B_1}{2}\right)^2 \left[\frac{e^{\mathbb{B}/2} - e^{-\mathbb{B}/2}}{e^{\mathbb{B}/2} + e^{-\mathbb{B}/2}}\right] = \text{Signal}(N,\text{A}).$$

One can see that the signal from *N*/2 of AX-systems, N/2 of A_2-system, and *N* of A-systems is the same. This is another demonstration of the validity of the Theorem, and confirmation of the fact that groupling of spins does not change their observable signal at HF.

One spin-1 (A). In the system of one spin-1 particles (A-system), there are only two single-quantum transitions, and they have the same intensity:

$$\langle 1,-1|\hat{I}_x|1,0\rangle = \langle 1,0|\hat{I}_x|1,1\rangle = \left\langle 1,0\left|\frac{\hat{I}_-}{2}\right|1,1\right\rangle = \frac{1}{\sqrt{2}}. \quad \text{(A6.10)}$$

This case is very similar to the case of two equivalent spins-½ (eq. A6.7) except for the lack of the singlet state. Thus, the total NMR signal from *N* spins-1 is a sum of two signals from the two allowed transitions:

$$\text{Signal}(N,A) \sim N \cdot (W_{+1\to 0} + W_{0\to -1}) \sim \left(\frac{\gamma\hbar B_1}{\sqrt{2}}\right)^2 [\,(n_{+1} - n_0) + (n_0 - n_{-1})] = \quad \text{(A6.11)}$$

$= \frac{N}{2}(\gamma\hbar B_1)^2\left[\frac{e^{\mathbb{B}}-e^{-\mathbb{B}}}{e^{\mathbb{B}}+1+e^{-\mathbb{B}}}\right]\xrightarrow{\mathbb{B}\to 0} N(\hbar\gamma B_1)^2 \cdot \frac{1}{3}\mathbb{B}.$

Thus, the receptivity of NMR detection at HF concerning spins-1 is 8/3 higher than that of spins-½, considering the same gyromagnetic ratio and spin number density. This result also matches the ratio of magnetizations obtained before (eq. A5.4).

A7. Solution to the Problem 3

We will first consider HT approximation case, and then a general case.

1. HT approximation.

Magnetization of *N* spins-*I* at thermal equilibrium is

$$m_z^{\{I\},N} = N \cdot (\gamma\hbar I)\frac{I+1}{3}\mathbb{B}. \tag{A7.1}$$

From the **Theorem 1** we can find the magnetization of a pair of spins:

$$m_z^{\{\frac{1}{2},\frac{1}{2}\},N} = 2m_z^{\{\frac{1}{2}\},N} = \frac{1}{2}N\gamma\hbar\mathbb{B}. \tag{A7.2}$$

For two-spin-½ systems at HT, all states are almost equally populated; hence, ¾N quasi-particles have spin-1 (triplet spin pairs), and ¼*N* have spin-0 (singlet spin pairs). Now lets calculate magnetization as a superposition of these two ansembles of particles:

$$m_z^{\{1\},\frac{3}{4}N,\mathrm{HT}} + m_z^{\{0\},\frac{1}{4}N,\mathrm{HT}} = \frac{3}{4}m_z^{\{1\},N,\mathrm{HT}} = \frac{3}{4}N\frac{2\gamma\hbar}{3}\mathbb{B} = \frac{1}{2}N\gamma\hbar\mathbb{B}. \tag{A7.3}$$

Hence, magnetization in this case can be calculated as a superposition of quasi-particles. Interestingly, the loss of total magnetization due to the population of the unobservable singlet states is compensated by the "stronger-magnetized" triplet manifolds.

2. General case. Let us now consider the general case of arbitrary numbers of equivalent spins-*I*. These spins can be grouped in a set of states associated with the total spin J such that each state from the set can be obtained using the rising or lowering operators of total spin (**Figure A1**). We will call each such a set of states a quasi-particle with spin- J as it can be described by its own orthogonal basis independent from other sets and it has all spin projections from J to $-J$. By definition, magnetization of the system is as follows:

$$m_z^{\{I_1 I_2 \dots\},N} = \frac{1}{\mathbb{Z}}N\gamma\hbar\sum_{\{I_{1\mathrm{z}}I_{2\mathrm{z}}\dots\}}(I_{1\mathrm{z}} + I_{2\mathrm{z}}\dots)e^{(I_{1\mathrm{z}}+I_{2\mathrm{z}}\dots)\mathbb{B}}. \tag{A7.4}$$

Here, the summation goes over all combinations of spin projections of individual spins. Using, however, the total spin basis, it can be written as

$$m_z^{\{I_1 I_2 \dots\},N} = \frac{1}{\mathbb{Z}}N\gamma\hbar\sum_J\sum_{J_\mathrm{z}=J}^{-J}J_\mathrm{z}e^{J_\mathrm{z}\mathbb{B}}. \tag{A7.5}$$

The last two equations are identical and differ only by how spin states are grouped. The equality (A.7.5) already gives a hint to the problem's solution. The statistical sum for each

quasiparticle with spin- J is $\mathbb{Z}_J = \sum_{J_z=J}^{-J} e^{J_z \mathbb{B}}$. Let us now introduce it in the eq. (A7.5) as follows:

$$m_z^{\{I_1 I_2 \dots\},N} = \sum_J \frac{\mathbb{Z}_J}{\mathbb{Z}} N \frac{\sum_{J_z=J}^{-J} \gamma \hbar J_z e^{J_z \mathbb{B}}}{\mathbb{Z}_J} = \sum_J \frac{\mathbb{Z}_J}{\mathbb{Z}} m_z^{\{J_z\},N} = \sum_J \rho_J m_z^{\{J_z\},N} = \sum_J m_z^{\{J_z\},N\rho_J}. \quad \text{(A7.6)}$$

Here, $\rho_J = \mathbb{Z}_J/\mathbb{Z}$ is a statistical fraction of realizing a particular total spin-J out of all possible total spins. Hence, indeed, one can calculate the magnetization of equivalent spins as a superposition of magnetizations comprising pseudo particles.

A8. Calculation of polarization of arbitrary coupled spin-*I* systems. Part 3: Proof of the equivalent formulation of the theorem (eq. 32)

In the main text, we derived $P_{rz}^{\{I_1 I_2 \dots I_n\},\mathrm{HT}}$ (eq. 31). Now lets find it without HT approximation and demonstrate that $P_{rz}^{\{I_1 I_2 \dots I_n\}} = P_z^{\{I_r\}}$ at thermal equilibrium. Polarization of spin-I_r as in a group of *n*-spins is

$$P_{rz}^{\{I_1 I_2 \dots I_n\}} = \frac{1}{I_r} \mathrm{Tr}\left(\hat{I}_{rz}^{\{I_1 I_2 \dots I_n\}} \hat{\rho}^{\{I_1 I_2 \dots I_n\}}\right) = \frac{1}{I_r} \frac{\mathrm{Tr}\left(\hat{I}_{rz}^{\{I_1 I_2 \dots I_n\}} \prod_m e^{\mathbb{B}^m \hat{I}_{mz}^{\{I_1 I_2 \dots I_n\}}}\right)}{\mathrm{Tr}\left(\prod_m e^{\mathbb{B}^m \hat{I}_{mz}^{\{I_1 I_2 \dots I_n\}}}\right)} \quad \text{(A8.1)}$$

When working with density matrices following equalities are very useful and will be used below often:

$$\mathrm{Tr}(A \otimes B) = \mathrm{Tr}(A)\mathrm{Tr}(B), \quad \text{(A8.2)}$$

for any square matrices A and B. Additionally we needed the mixed-product property of the direct product operator:

$$(A \otimes B) \cdot (C \otimes D) = A \cdot C \otimes B \cdot D. \quad \text{(A8.3)}$$

where A, B, C, and D are matrices of the appropriate size for scalar (dot) product.

Lets simplify denominator in eq. A8.1:

$$\mathrm{Tr}\left(\prod_m e^{\mathbb{B}^m \hat{I}_{mz}^{\{I_1 I_2 \dots I_n\}}}\right) = \mathrm{Tr}\left(\prod_m e^{\mathbb{B}^m (\hat{1}_1 \otimes \dots \hat{1}_{r-1} \otimes \hat{I}_{mz} \otimes \hat{1}_{r+1} \dots \otimes \hat{1}_n)}\right) = \quad \text{(A8.4)}$$
$$= \mathrm{Tr}\left(\otimes_m e^{\mathbb{B}^m \hat{I}_{mz}}\right) = \prod_{m=1}^N \mathrm{Tr}\left(e^{\mathbb{B}^m \hat{I}_{mz}}\right).$$

Nominator equals to

$$\mathrm{Tr}\left(\hat{I}_{rz}^{\{I_1 I_2 \dots I_n\}} \prod_m e^{\mathbb{B}^m \hat{I}_{mz}^{\{I_1 I_2 \dots I_n\}}}\right) = \frac{d}{d(\mathbb{B}^r)} \mathrm{Tr}\left(\prod_m e^{\mathbb{B}^m \hat{I}_{mz}^{\{I_1 I_2 \dots I_n\}}}\right) = \frac{d}{d(\mathbb{B}^r)} \prod_{m=1}^N \mathrm{Tr}\left(e^{\mathbb{B}^m \hat{I}_{mz}}\right) = \quad \text{(A8.5)}$$
$$= \frac{\prod_{m=1}^N \mathrm{Tr}\left(e^{\mathbb{B}^m \hat{I}_{mz}}\right)}{\mathrm{Tr}\left(e^{\mathbb{B}^r \hat{I}_{rz}}\right)} \frac{d\mathrm{Tr}\left(e^{\mathbb{B}^r \hat{I}_{rz}}\right)}{d(\mathbb{B}^r)} = \frac{\prod_{m=1}^N \mathrm{Tr}\left(e^{\mathbb{B}^m \hat{I}_{mz}}\right)}{\mathrm{Tr}\left(e^{\mathbb{B}^r \hat{I}_{rz}}\right)} \mathrm{Tr}\left(\hat{I}_{rz} e^{\mathbb{B}^r \hat{I}_{rz}}\right).$$

From eq. (4.1-3) it follows that

$$P_{rz}^{\{I_1I_2\ldots I_n\}} = \frac{1}{I_r}\frac{\mathrm{Tr}\left(\hat{I}_{rz}^{\{I_1I_2\ldots I_n\}}\prod_m e^{\mathbb{B}^m\hat{I}_{mz}^{\{I_1I_2\ldots I_n\}}}\right)}{\mathrm{Tr}\left(\prod_m e^{\mathbb{B}^m\hat{I}_{mz}^{\{I_1I_2\ldots I_n\}}}\right)} = \frac{\mathrm{Tr}\left(\hat{I}_{rz}e^{\mathbb{B}^r\hat{I}_{rz}}\right)}{\mathrm{Tr}\left(e^{\mathbb{B}^r\hat{I}_{rz}}\right)} = P_{\mathrm{z}}^{\{I_r\}}. \tag{A8.6}$$

Hence, the statement was proved: the polarization of a spin is system-size independent because, on the right-hand side, there is no information regarding the size of the system at HF-approximation.

A9. Quadrupolar polarization

To find "quadrupolar polarization" (eq. 41), $\hat{P}_{\mathrm{Q}}^{\{I\}} = \frac{3(\hat{I}_{\mathrm{z}})^2 - I(I+1)\hat{1}}{I^2}$, we need to know the following quantity: $\mathrm{Tr}\left(\left(\hat{I}_{\mathrm{z}}\right)^2 e^{\mathbb{B}\hat{I}_{\mathrm{z}}}\right)$ and $\mathrm{Tr}\left(e^{\mathbb{B}\hat{I}_{\mathrm{z}}}\right)$. We will use the same approach as before in appendix 1. We already derived $\mathrm{Tr}\left(e^{\mathbb{B}\hat{I}_{\mathrm{z}}}\right)$ (eq A1.1) and $\frac{d}{d\mathbb{B}}\mathrm{Tr}\left(e^{\mathbb{B}\hat{I}_{\mathrm{z}}}\right) = \mathrm{Tr}\left(\hat{I}_{\mathrm{z}}e^{\mathbb{B}\hat{I}_{\mathrm{z}}}\right)$ (eq A1.2) and now we will find $\mathrm{Tr}\left(\left(\hat{I}_{\mathrm{z}}\right)^2 e^{\mathbb{B}\hat{I}_{\mathrm{z}}}\right)$:

$$\frac{d^2}{d\mathbb{B}^2}\mathrm{Tr}\left(e^{\mathbb{B}\hat{I}_{\mathrm{z}}}\right) = \mathrm{Tr}\left(\left(\hat{I}_{\mathrm{z}}\right)^2 e^{\mathbb{B}\hat{I}_{\mathrm{z}}}\right) = \tag{A9.1}$$
$$= \frac{e^{\left(I+\frac{1}{2}\right)\mathbb{B}} - e^{-\left(I+\frac{1}{2}\right)\mathbb{B}}}{e^{\frac{\mathbb{B}}{2}} - e^{-\frac{\mathbb{B}}{2}}}\left(\left(I+\frac{1}{2}\right)^2 + \frac{1}{4} + \frac{1}{2\sinh^2\left(\frac{\mathbb{B}}{2}\right)}\right) - \left(I+\frac{1}{2}\right)\frac{e^{\left(I+\frac{1}{2}\right)\mathbb{B}} + e^{-\left(I+\frac{1}{2}\right)\mathbb{B}}}{e^{\frac{\mathbb{B}}{2}} - e^{-\frac{\mathbb{B}}{2}}}\coth\left(\frac{\mathbb{B}}{2}\right).$$

Therefore, combining eq A1.1, A1.2, and eq. 41 quadrupolar polarization can be found

$$P_{\mathrm{Q}}^{\{I\}} = \frac{3\mathrm{Tr}\left((\hat{I}_{\mathrm{z}})^2 e^{\mathbb{B}\hat{I}_{\mathrm{z}}}\right) - I(I+1)}{I^2\mathrm{Tr}\left(e^{\mathbb{B}\hat{I}_{\mathrm{z}}}\right)} = \frac{2(I+1)}{I} + \frac{3}{2I^2} + \frac{3}{2I^2\sinh^2\left(\frac{\mathbb{B}}{2}\right)} - \frac{3}{I^2}\left(I+\frac{1}{2}\right)\coth\left(\left(I+\frac{1}{2}\right)\mathbb{B}\right)\coth\left(\frac{\mathbb{B}}{2}\right). \tag{A9.2}$$

For example, for spin-1 particles, the quadrupolar polarization is

$$P_{\mathrm{Q}}^{\{1\}} = \frac{11}{2} + \frac{3}{2\sinh^2\left(\frac{\mathbb{B}}{2}\right)} - \frac{9}{2}\coth\left(\frac{3}{2}\mathbb{B}\right)\coth\left(\frac{\mathbb{B}}{2}\right). \tag{A9.3}$$

A10. Constraints on the elements of the density matrix of two spin-½

Let us look at the diagonal elements in density matrix, $\hat{\rho}^{\{½½\}}$, in Zeeman basis when only net magnetization of each spin and a two-spin order (eq. 37) are present in the system. All diagonal elements (populations) must be real numbers between 0 and 1:

$$\begin{aligned} 1 &\geq \rho_{\alpha\alpha} = \frac{1}{4} + \frac{1}{4}P_{1\mathrm{z}}^{\{½\}} + \frac{1}{4}P_{2\mathrm{z}}^{\{½\}} + \frac{1}{4}P_{\mathrm{zz}}^{\{½½\}} \geq 0, \\ 1 &\geq \rho_{\alpha\beta} = \frac{1}{4} + \frac{1}{4}P_{1\mathrm{z}}^{\{½\}} - \frac{1}{4}P_{2\mathrm{z}}^{\{½\}} - \frac{1}{4}P_{\mathrm{zz}}^{\{½½\}} \geq 0, \\ 1 &\geq \rho_{\beta\alpha} = \frac{1}{4} - \frac{1}{4}P_{1\mathrm{z}}^{\{½\}} + \frac{1}{4}P_{2\mathrm{z}}^{\{½\}} - \frac{1}{4}P_{\mathrm{zz}}^{\{½½\}} \geq 0, \\ 1 &\geq \rho_{\beta\beta} = \frac{1}{4} - \frac{1}{4}P_{1\mathrm{z}}^{\{½\}} - \frac{1}{4}P_{2\mathrm{z}}^{\{½\}} + \frac{1}{4}P_{\mathrm{zz}}^{\{½½\}} \geq 0. \end{aligned} \tag{A10.1}$$

These 8 inequalities can be written in the matrix form as

$$3 \geq \begin{pmatrix} +1 & +1 & +1 \\ +1 & -1 & -1 \\ -1 & +1 & -1 \\ -1 & -1 & +1 \end{pmatrix} \begin{pmatrix} P_{1z}^{\{½\}} \\ P_{2z}^{\{½\}} \\ P_{zz}^{\{½½\}} \end{pmatrix} \geq -1. \quad \text{(A10.2)}$$

All conditions "3 ≥" are fulfilled automatically because all polarization values are between -1 and 1. Sometimes it is useful to quantify polarization as an average net polarization, $P_{\mathrm{net}} = \frac{P_{1z}^{\{½\}} + P_{2z}^{\{½\}}}{2}$, antiphase polarization, $P_{\mathrm{anti}} = \frac{P_{1z}^{\{½\}} - P_{2z}^{\{½\}}}{2}$, and zz-spin order, $P_{\mathrm{zz}} = P_{\mathrm{zz}}^{\{½½\}}$, of the two-spin system. Then, the density matrix and restrictions can be written as

$$\hat{\rho}^{\{½½\}} = \frac{\hat{1}}{4} + \frac{1}{2} P_{\mathrm{net}} \left(\hat{I}_{1z}^{\{½½\}} + \hat{I}_{2z}^{\{½½\}} \right) + \frac{1}{2} P_{\mathrm{anti}} \left(\hat{I}_{1z}^{\{½½\}} - \hat{I}_{2z}^{\{½½\}} \right) + P_{\mathrm{zz}} \hat{I}_{1z}^{\{½½\}} \hat{I}_{2z}^{\{½½\}}, \quad \text{(A10.3)}$$
$$\begin{pmatrix} 2 & 0 & +1 \\ 0 & 2 & -1 \\ 0 & -2 & -1 \\ -2 & 0 & +1 \end{pmatrix} \begin{pmatrix} P_{\mathrm{net}} \\ P_{\mathrm{anti}} \\ P_{\mathrm{zz}} \end{pmatrix} \geq -1.$$

From eq. (A10.3) one can get the following 8 conditions (**Figure 5A**):

$$\begin{gathered} 1 \geq 0.5 P_{\mathrm{zz}} + 0.5 \geq P_{\mathrm{net}} \geq -0.5 P_{\mathrm{zz}} - 0.5 \geq -1, \\ 1 \geq -0.5 P_{\mathrm{zz}} + 0.5 \geq P_{\mathrm{anti}} \geq 0.5 P_{\mathrm{zz}} - 0.5 \geq -1, \\ 1 \geq 1 - 2|P_{\mathrm{anti}}| \geq P_{\mathrm{zz}} \geq -1 + 2|P_{\mathrm{net}}| \geq -1. \end{gathered} \quad \text{(A10.4)}$$

One can now see that there are restrictions on P_{anti} and P_{net} to be less than 1, the sum of their absolute values should be also smaller than one: $1 \geq |P_{\mathrm{net}}| + |P_{\mathrm{anti}}|$.

If P_{anti} = 0 then eq. A11.4 (**Figure 4B**) simplifies to

$$\begin{gathered} 1 \geq 0.5 P_{\mathrm{zz}} + 0.5 \geq P_{\mathrm{net}} \geq -0.5 P_{\mathrm{zz}} - 0.5 \geq -1, \\ 1 \geq P_{\mathrm{zz}} \geq -1 + 2|P_{\mathrm{net}}| \geq -1. \end{gathered} \quad \text{(A10.5)}$$
